# Cerebral cavernous malformations: epidemiology, molecular pathogenesis, diagnosis and therapy

**DOI:** 10.1186/s43556-026-00619-w

**Published:** 2026-10-10

**Authors:** Suyun Yu, Weiwei Zheng, Yanhong Pan, Liwenyu Chen, Jing Ma, Mengyuan Gao, Wei Zou, Yin Lu, Yang Zhao

**Affiliations:** 1 Jiangsu Key Laboratory for Pharmacology and Safety Research of Chinese Materia Medica, Nanjing University of Chinese Medicine, Nanjing, 210023 China; 2 State Key Laboratory of Mechanism and Quality of Chinese Medicine, Macau University of Science and Technology, Macau, 999078 China; 3 School of Medicine & Holistic Integrative Medicine, Nanjing University of Chinese Medicine, Nanjing, 210023 China; 4 Jiangsu Joint International Research Laboratory of Chinese Medicine and Regenerative Medicine, Nanjing University of Chinese Medicine, Nanjing, 210023 China

**Keywords:** CCM, Epidemiology, Molecular pathogenesis, Diagnosis, Therapy

## Abstract

Cerebral cavernous malformations (CCMs) are cerebrovascular diseases characterized by vascular lesions of the central nervous system (CNS) that can result in epileptic seizures, headaches, focal neurologic deficits (FNDs), and hemorrhagic stroke. Germline and somatic loss-of-function (LOF) mutations in one of the three established CCM-associated genes, namely *CCM1* (or krev interaction trapped protein 1, *KRIT1*), *CCM2*, and *CCM3* (or programmed cell death 10, *PDCD10*), which have been validated as core drivers of CCM pathogenesis and progression. Over the past few decades, a substantially improved understanding of the hallmarks and molecular basis of CCMs has been achieved. Key pathological hallmarks include but are not limited to excessive angiogenesis, vascular leakage, endothelial-mesenchymal transition (EndMT), defective autophagy, inflammation and immune response, oxidative stress, influenced gut microbiome and altered brain vascular microenvironment. These hallmarks are orchestrated by dysregulated signaling cascades, notably MEKK3-KLF2/4, PI3K-mTOR, and RhoA-ROCK, which integrate genetic, inflammatory, and hemodynamic cues. Concurrently, sophisticated neuroimaging (MRI with SWI/QSM) and genetic testing now enable precise diagnosis and risk stratification. Although surgical resection remains the mainstay for accessible symptomatic lesions, a wave of mechanism‑based pharmacological candidates has shown promising efficacy in preclinical models, and several have advanced to early‑phase clinical trials. This review summarizes the current understanding of CCM epidemiology, molecular pathogenesis, diagnosis and therapy, highlighting how the evolving multi‑hit concept and the dissection of downstream signaling are paving the way towards personalized management of this complicated vascular disease.

## Introduction

Cerebral vascular malformations are grouped into four main categories including arteriovenous, venous, capillary, and cavernous malformations [1–3]. Among them, Cerebral Cavernous Malformations (CCMs) are a frequent type of vascular malformation predominantly in the brain with a prevalence of approximately 0.5% [2, 3]. CCMs primarily occur in adulthood, they are accepted to be a major driving force of hemorrhagic stroke and is characterized by abnormal clusters of dilated and fragile capillaries with mulberry-like structures [4, 5]. In general, CCMs are categorized as sporadic CCM (sCCM) or familial CCM (fCCM) diseases and lined by a single layer of endothelial cells (ECs) with disrupted EC cell–cell junctions. Of note, the majority of CCM patients do not exhibit clinical manifestations, hence only a small percentage of these patients can be identified incidentally [6]. Almost 80% of CCM patients occur as a sporadic disease with one lesion, while 20% of them tend to emerge as a familial autosomal dominant disorder commonly accompanied by more than one lesion [7–9]. In the past few years, the genetics and functions of CCM genes including *CCM1*, *CCM2* and *CCM3* have been gaining increasing attention as involved in the progression of CCM, and the majority of CCM patients carry a heterozygous germline loss-of-function (LOF) variant in any one of these three genes from both fCCM and sCCM cases [10–12]. It has been well accepted that various known fundamental mutations in the CCM genes that are responsible for a large proportion of fCCM cases. For instance, NM_194456.1 (*KRIT1*): c.1363C > T(p.Gln455Ter) is found in the majority of Hispanic American cases of fCCM [13–15]. Furthermore, *CCM1* mutations are the most common cause of familial CCMs, followed by *CCM2* and *CCM3* [5, 16–18]. CCM1, CCM2, or CCM3 as three classic CCM proteins encoded by genes are present in the same complex formed within the cell [19], the functional deficiency of any one of the three protein is validated to be the molecular engine that eventually drives the initiation of CCM disease [20]. Nevertheless, LOF of CCM3 in either humans or mice frequently leads to more severe conditions, which are ascribed to the functions of CCM3 in the gut epithelium that are not shared with CCM1 or CCM2 [16, 17, 21, 22].

**Fig. 1:**
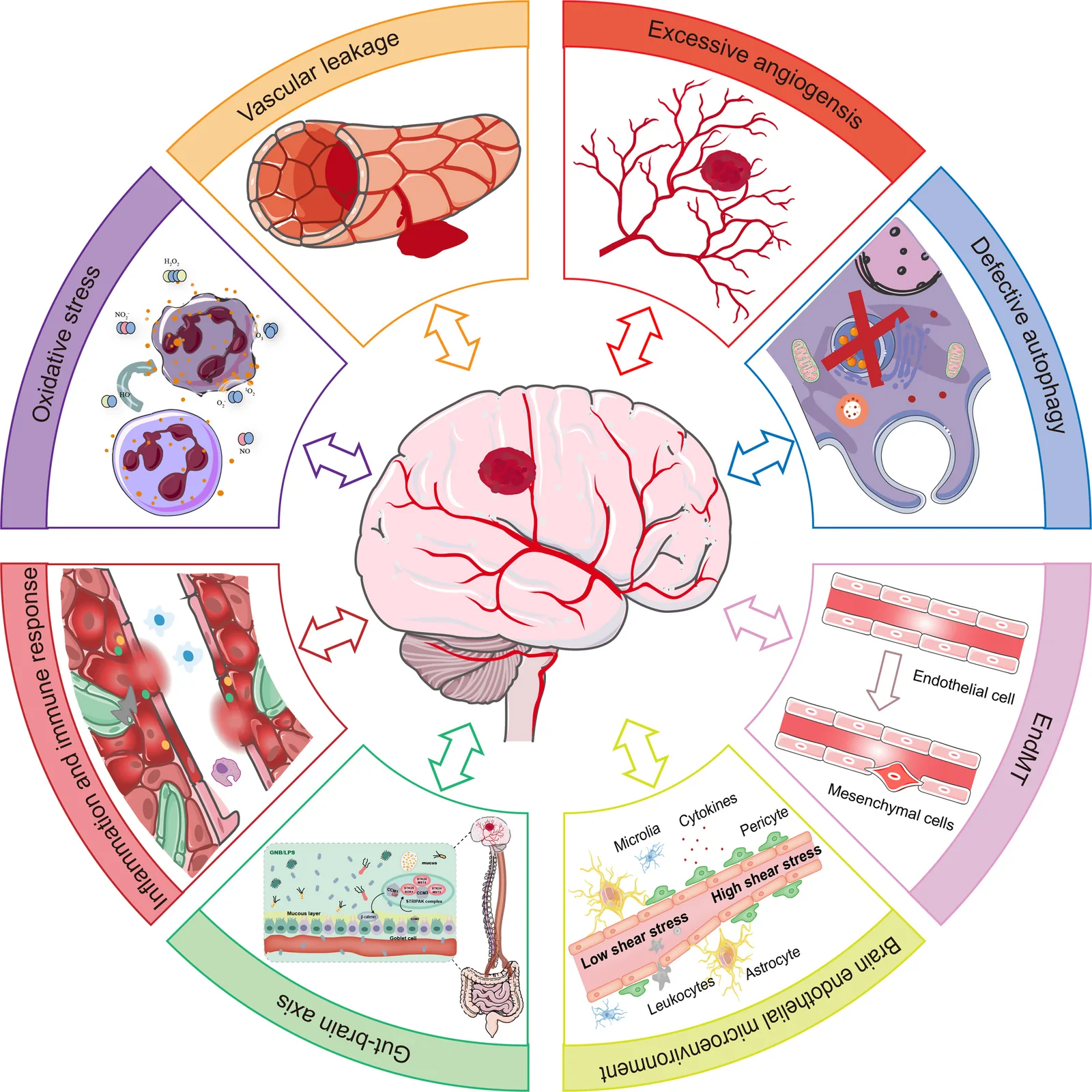
The hallmarks of CCMs. Schematic illustration for the eight hallmark capabilities shown during the progression of CCMs. The past few years have witnessed substantial progress on understanding the mechanistic underpinnings of each hallmark

Over the past decade, the understanding of CCM pathogenesis has evolved from a simple “two-hit” model (germline plus somatic mutation) to a “multi-hit” framework, where somatic mutations in non-CCM genes and environmental factors influence disease severity [23–27]. This complexity is further underscored by eight interrelated pathological hallmarks (Fig. 1): excessive angiogenesis, vascular leakage, endothelial-mesenchymal transition (EndMT), defective autophagy, inflammation and immune response, oxidative stress, influenced gut microbiome and altered brain vascular microenvironment. While neuroimaging techniques including magnetic resonance imaging (MRI) with susceptibility-weighted imaging (SWI)/quantitative susceptibility mapping (QSM) and genetic testing have enabled precise diagnosis and risk stratification [28–30], and surgical resection remains the mainstay for accessible symptomatic lesions [31, 32], critical knowledge gaps persist, such as the temporal dynamics of “multi-hit” mutations, the context-dependent roles of EndMT, and the translational gap between preclinical therapeutic targets and clinical efficacy. These knowledge gaps motivate this review, whose scope, structure, and key messages are summarized below.

This review synthesizes current evidence from cell-based models, animal studies, and clinical cohorts to offer a comprehensive update on CCM epidemiology, molecular pathogenesis, diagnostic strategies, and therapeutic approaches. We began with outlining the epidemiological landscape of CCMs, including prevalence, demographic patterns, genetic susceptibility, and modifiable risk factors. Subsequently, the molecular pathogenesis was summarized accordingly, moving beyond the canonical genetic framework to explore how the CCM signaling complex integrates diverse inputs and how each pathological hallmark contributes to disease progression. We then reviewed contemporary diagnostic modalities of CCMs, emphasizing the synergistic integration of advanced neuroimaging with molecular genetic testing. Finally, therapeutic strategies were comprehensively listed, ranging from surgical management to emerging mechanism-based pharmacotherapies, highlighting both preclinical advances and ongoing clinical trials. By synthesizing the interdependence of genetic drivers, signaling pathways, and pathological hallmarks, this review aims to inform clinical decision-making and identify promising directions for future research toward personalized management of this complicated vascular disease.

## Epidemiology

A precise understanding of the epidemiology of CCMs is essential for appreciating its public health impact and guiding research into its etiology. While initially thought to be a rare curiosity, the prevalence of CCMs is higher than many other inherited neurological disorders, and its clinical presentation is remarkably heterogeneous. This section provides an overview of CCM prevalence and incidence, key demographic patterns, genetic architecture of the disease, and associated risk factors that may influence its development.

### Prevalence and incidence

CCMs are regarded as the third most common cerebral vascular malformation, following developmental venous anomalies and capillary telangiectasias. Autopsy and imaging-based studies have established a prevalence of approximately 0.5% in the general population [2, 3, 33, 34]. However, this figure likely underestimates the true burden of the disease. A significant proportion of individuals with CCMs, estimated to be between 50 and 70%, remain asymptomatic throughout their lives, with lesions frequently discovered incidentally during neuroimaging for unrelated reasons [6, 35, 36]. The widespread availability of MRI has led to a dramatic increase in incidental diagnosis, which now accounts for up to 50% of all identified cases [35, 37]. Of note, the symptomatic prevalence is much lower, estimated at 0.037% [38]. Another retrospective analysis of 199 patients with sporadic solitary CCMs reported an overall annual hemorrhage rate of 6.03%, with higher rates (11.95%) in those presenting with prior hemorrhage [39]. These findings underscore the importance of distinguishing between incidentally detected and symptomatic lesions when estimating population-level burden. The annual incidence of first clinical presentation of CCMs (e.g., hemorrhage or seizure) is estimated at 0.56 per 100,000 person-years, with the incidence of first hemorrhage being slightly lower at 0.31 per 100,000 person-years [4, 40]. A recent retrospective cohort study focusing on newly diagnosed CCMs in Finland reveals that CCM incidence is higher than previously reported and has exhibited a progressive increase over the study period [41], with these lesions imposing a considerable and growing burden on healthcare systems.

### Demographic patterns

CCMs affect individuals of all ages, from neonates to the elderly, with no significant overall sex predilection, though lesion burden tends to increase with age [38, 42]. A retrospective analysis of 199 patients with sCCM reported that age < 45 years was an independent risk factor for hemorrhage, suggesting young patients may have more aggressive phenotypes [39]. Another study of 105 CCM patients found a positive correlation between mean lesional susceptibility (a marker of iron deposition) and patient age, with a 4.1% increase in susceptibility per decade of life [43]. This age-related increase in iron deposition might reflect cumulative hemorrhage events, contributing to the higher symptomatic burden observed in older patients. Additionally, a case series of patients with CCMs who received intravenous tissue plasminogen activator (IV-tPA) for acute ischemic stroke suggested a higher incidence of symptomatic intracerebral hemorrhage (ICH), particularly in women and in patients with calcified lesions [44], although these observational findings remain anecdotal and require confirmation in larger studies. Age-dependent clinical presentations are evident in pediatric CCMs, with infants more commonly exhibiting elevated intracranial pressure and isolated lesions compared to older children [45]. Ethnic and geographic variations in CCM prevalence have also been noted. Certain ethnic populations, particularly Hispanic-American families, exhibit a higher prevalence of fCCM due to specific founder mutations, most notably the common Hispanic mutation in *KRIT1* (*CCM1*; p.Gln455Ter, Q455X) [46, 47]. A study of 188 Hispanic CCM patients harboring the founder *KRIT1* mutations demonstrated that genetic regulators, such as cytochrome P450 (CYP) and matrix metalloproteinase (MMP) polymorphisms, influenced lesion severity [48, 49]. Indeed, the *CYP4F12* rs11085971 variant was associated with a higher number of large lesions, while *MMP3* rs117153070 was related to increased ICH risk. These findings suggest that ethnic-specific genetic backgrounds may govern CCM phenotypes, emphasizing the need for population-tailored risk stratification.

### Genetic susceptibility and inheritance patterns

**Fig. 2:**
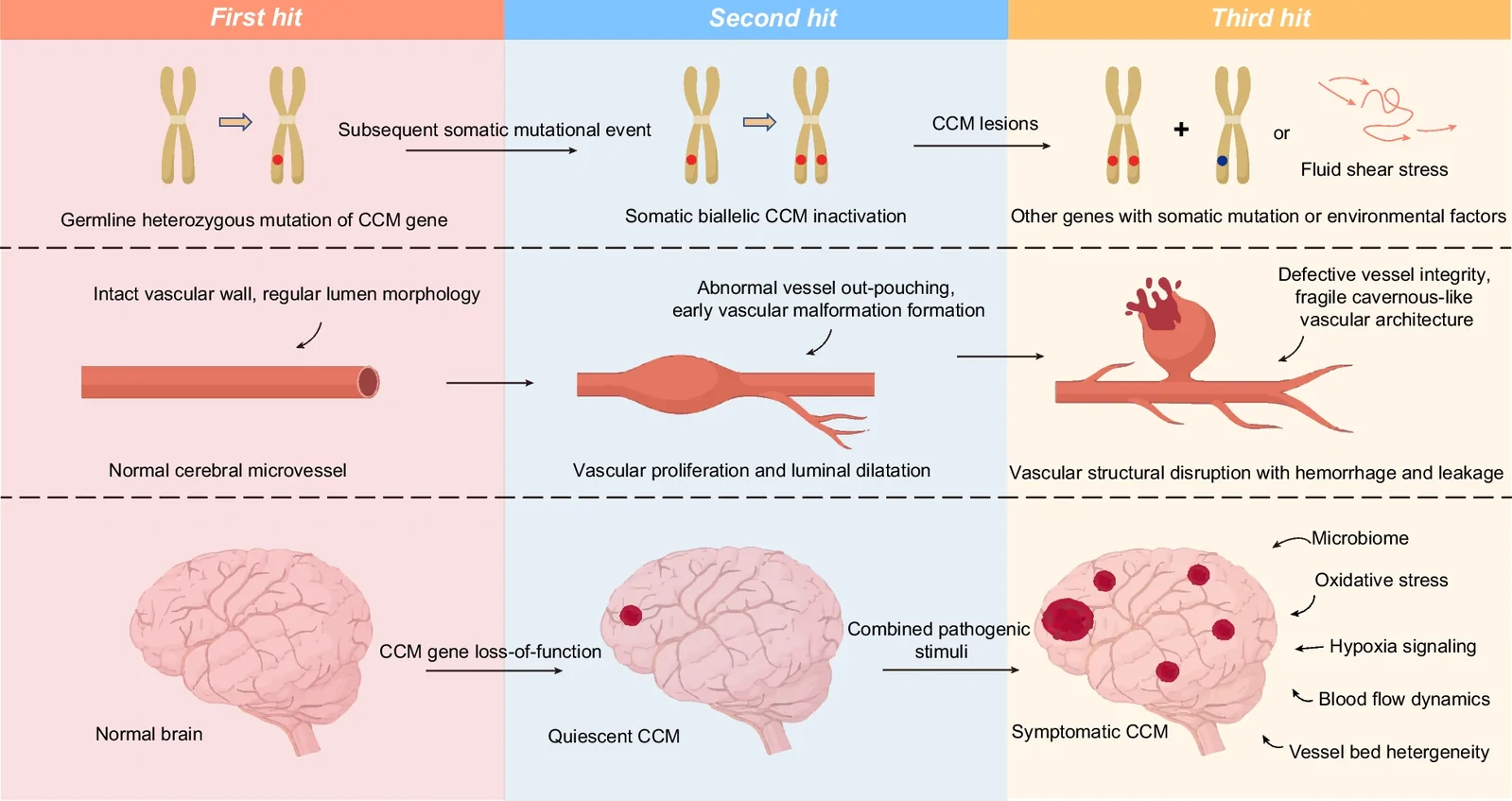
A conceptual multi-hit model of CCM pathogenesis. CCM lesion formation is generally initiated by biallelic loss of a CCM gene, but the number, type, and temporal order of subsequent hits vary across CCM subtypes. Left: In fCCMs and some multiple-lesion cases, a heterozygous germline LOF variant in *KRIT1* (*CCM1*), *CCM2*, or *PDCD10* (*CCM3*) acts as the first hit, resulting in haploinsufficiency in ECs. In solitary sCCMs, both hits may instead be somatic. Middle: A second somatic LOF variant in the remaining wild-type allele causes biallelic CCM-gene inactivation in individual ECs, which may undergo clonal expansion and initiate lesion formation. Right: Additional somatic variants in genes such as *MAP3K3* or *PIK3CA*, together with hemodynamic and microenvironmental factors, may further promote lesion growth and phenotypic heterogeneity. These events can contribute to the development of characteristic cavernous vasculature, vascular leakage, and microhemorrhage. This framework is presented as a conceptual synthesis rather than a unified mechanism applicable to all CCM subtypes; the relative contribution and temporal sequence of individual hits remain to be determined

In general, CCMs are categorized as sCCM or fCCM diseases and lined by a single layer of ECs with disrupted EC cell–cell junctions. The two major clinical forms of CCM differ in their genetic architecture, fCCM follows an autosomal dominant inheritance pattern with incomplete penetrance and variable expressivity. Therefore, not all carriers develop lesions, and lesion number and clinical severity can vary substantially even within the same family [7–9]. To date, heterozygous germline LOF mutations in one of three genes have been identified as the cause of fCCM: *CCM1*, *CCM2*, and *CCM3*. Mutations in *CCM1* are the most common, found in 40–70% of familial cases, followed by *CCM2* (15–20%) and *CCM3* (10–15%) [5, 16–18]. Genotype–phenotype correlations have emerged, individuals with *CCM1* mutations are inclined to be relevant to cutaneous vascular lesions [50, 51], and individuals with a mutation in *CCM2* tend to be asymptomatic and harbor a relatively small number of lesions [16]. Nevertheless, LOF of *CCM3* in either humans or mice frequently leads to more severe conditions, which are ascribed to the functions of CCM3 in the gut epithelium that are not shared with CCM1 or CCM2 [16, 17, 21, 22]. For decades, the etiology of sCCM was unknown. However, pioneering work has demonstrated that the vast majority of these sporadic lesions also harbor biallelic somatic mutations in one of the three CCM genes within the lesion’s ECs, perfectly mirroring the “two-hit” mechanism of tumorigenesis proposed by Knudson and now established as central to CCM pathogenesis [23, 52, 53] (Fig. 2).

### Associated risk factors

While genetic predisposition remains the primary driver of CCM formation, accumulating evidence highlights the significant roles of environmental and external factors as regulators of disease progression. The most well-established environmental risk factor is therapeutic cranial irradiation. Patients who undergo radiation therapy for brain tumors-especially during childhood-exhibit a markedly increased risk of developing de novo CCMs within the radiation field, often decades after treatment [54, 55]. Histologically, these radiation-induced lesions are indistinguishable from their genetic counterparts and also harbor somatic mutations in CCM genes, indicating that radiation may accelerate the somatic “second-hit” mutational event in ECs. Other environmental factors including oxidative stress, inflammation, and hemodynamic forces are now recognized as critical contributors to both lesion initiation and progression [56]. Notably, the influence of hemodynamic factors appears to be complicated. For example, some studies suggest that higher systolic blood pressure may correlate with fewer lesions, underscoring the nuanced role of biomechanical forces in the disease microenvironment [57]. Together, these observations reinforce that CCM development arises from an interplay between genetic susceptibility and modifiable environmental exposures. Other potential risk factors, such as hormonal influences (e.g., estrogen and progesterone), have been proposed but are less well-established [58], representing an important field for future epidemiological research.

## Molecular pathogenesis

The molecular pathogenesis of CCMs is a paradigm of how localized genetic hits in vascular ECs unleash a cascade of dysregulated signaling, leading to the hallmark structural and functional lesions. Moving beyond the established genetic framework, recent studies paint a picture of a dynamic, multi-hit process influenced by cellular microenvironment, inflammatory cues, and systemic factors. This section synthesizes these advances into a cohesive narrative centered around the core pathological hallmarks: excessive angiogenesis, vascular leakage, EndMT, defective autophagy, inflammation, oxidative stress, gut-brain axis involvement, and altered vascular microenvironment, while integrating critical appraisal of the evidence and highlighting unresolved controversies and translational implications. This hierarchical framework, from genotype to signaling dysregulation to cellular phenotypes to tissue pathology, distinguishes our review from previous summaries that present these elements in parallel.

### Genetic basis of CCMs: beyond the canonical “two-hit” model

The pathogenesis of CCMs is not explained by germline mutations alone. Significant clinical and radiological heterogeneity, even among family members carrying the same mutation, strongly supports the involvement of additional genetic or environmental factors. This has led to the evolution of multi-hit models of CCM pathogenesis. In addition to mutations in CCM genes, an array of other genes with somatic mutations have been described to be associated with excessive angiogenesis, such as phosphatidylinositol-4,5-bisphosphate 3-kinase catalytic subunit alpha (*PIK3CA*), mitogen-activated protein kinase kinase kinase 3 (*MAP3K3*, encoding the MEKK3 protein) and mitogen-activated protein kinase kinase 7 (*MAP2K7*), which are thought to be responsible for the triple-hit mode to instruct the progression of CCM disease [25–27] (Fig. 2). Recent studies have refined the genotype–phenotype landscape, showing that somatic *MAP3K3* and *PIK3CA* mutations define distinct lesion subtypes with differential risks of hemorrhage and epilepsy [59, 60]. This shift in understanding reframes CCMs not merely as a consequence of CCM complex disruption, but as a disease where the genetic landscape within ECs can be heterogeneous and progressive. A critical, often overlooked, point is the chronological order and clonal selection of these hits. Are *PIK3CA* mutations truly a “third hit” required after CCM gene biallelic loss, or can they occur concurrently, defining distinct molecular subtypes? The low allele frequency of *PIK3CA* GOF mutations in bulk sequencing (< 20%) [26] is consistent with an amplifier role, but variant allele frequency alone cannot establish temporal order. Functional studies support a model in which *MAP3K3* activation can initiate lesions in some contexts, whereas *PIK3CA* activation primarily enhances lesion growth [27, 61]. Furthermore, single-nucleus DNA sequencing has recently unveiled somatic loss-of-heterozygosity in *CCM1* or *CCM2* as an alternative genetic mechanism driving biallelic inactivation, explaining a subset of previously genetically unsolved CCM lesions [62]. Taken together, these observations support a conceptual “threshold” synthesis in which germline mutations are proposed to lower the threshold for lesion initiation, whereas somatic mutations may push ECs beyond this threshold. However, this model should be presented as a conceptual framework rather than a unified mechanism that applies equally to all CCM subtypes, and the precise chronological and causal relationships among the different genetic hits remain to be fully resolved.

### The CCM signaling complex: a central hub for vascular stability

The CCM proteins form a functional complex that acts as a key negative regulator of three interconnected signaling pathways: MEKK3-KLF2/4, PI3K-mTOR, and RhoA-ROCK. Dysregulation of these pathways, in concert with additional context-dependent inputs, contributes to rather than exclusively drives the diverse pathological features of CCMs. These pathways are not isolated but exhibit extensive crosstalk, with MEKK3-KLF2/4 serving as the upstream integrator of genetic, inflammatory, and hemodynamic cues, and PI3K-mTOR and RhoA-ROCK signaling as downstream effectors mediating specific cellular phenotypes.

The MEKK3-KLF2/4 axis is widely regarded as a major signaling node in CCM pathogenesis, although it operates within a broader, interconnected signaling network rather than as a single dominant pathway. CCM gene LOF releases MEKK3 from inhibitory constraints, leading to activation of the transcription factors KLF2 and KLF4 [61, 63]. These factors upregulate pro-angiogenic genes (e.g., *EGFL7*) and downregulate anti-angiogenic factors (e.g., *THBS1*, encoding TSP1), creating a pro-angiogenic imbalance [64, 65]. Additionally, MEKK3-KLF2/4 axis integrates external cues, low shear stress (a hallmark of brain microvasculature) and lipopolysaccharide (LPS) both enhance pathway activation, thereby explaining the brain tropism of lesions and the link between gut-brain axis and CCM progression [66–68]. Although MEKK3 inhibition effectively blocks CCM lesion formation in mouse models, its role in normal cardiovascular development raises concerns about off-target effects, highlighting the need for EC-specific or tissue-restricted targeting strategies [69, 70]. The PI3K-mTOR pathway is hyperactivated in CCM ECs due to both CCM LOF and KLF4 GOF, resulting in suppression of autophagy (via inhibition of ULK1) and promotion of EC proliferation [25, 71, 72]. Inhibitors of mTOR rescue autophagic flux and reverse CCM phenotypes in cellular and animal models, but clinical translation is hindered by systemic side effects [73, 74]. Notably, vitamin D, an accessible and low-toxicity mTOR repressor, reduces CCM lesion burden in preclinical models by restoring autophagy, suggesting vitamin D supplementation as a potential adjuvant strategy for CCM patients with low vitamin D levels, although direct clinical evidence in CCM patients is still lacking [75–77]. This connection between a nutritional factor and a core molecular pathway not only provides a translational opportunity but also underscores the interplay between environmental regulators and genetic susceptibility.

The RhoA-ROCK pathway mediates vascular leakage, a key early phenotype in CCMs. CCM2 normally interacts with Smurf1 to promote RhoA degradation [78]. CCM LOF impairs this process, leading to RhoA accumulation, ROCK activation, and phosphorylation of myosin light chain (pMLC) [79]. This increases EC contractility, disrupts tight junctions (TJs), and impairs the blood–brain barrier (BBB) [80]. The sequential disruption of TJs further implies that early intervention targeting TJs (e.g., Claudin-5 stabilizers) can prevent irreversible barrier damage and reduce hemorrhage risk, a strategy that aligns with the diagnostic goal of identifying pre-symptomatic lesions [81, 82]. The extent to which these pathways are recapitulated in the mature human cerebral vasculature, and their relative contribution to lesion initiation versus progression, remains an active area of investigation. The field is moving from viewing the complex as a simple “off-switch” for a single pathway to appreciating its role as a signaling integrator at the junctional hub.

### Cellular mechanisms and pathobiology: decoding the hallmark features

The eight hallmark features described below operate at distinct biological levels. The first four features, namely excessive angiogenesis, vascular leakage, EndMT, and defective autophagy, are core cellular and tissue phenotypes that directly drive lesion formation and progression. The remaining four, including inflammation, oxidative stress, gut microbiome dysregulation, and altered brain vascular microenvironment, are systemic and microenvironmental modifiers that amplify these core phenotypes and also arise as secondary consequences, creating a vicious cycle of disease progression.

#### Excessive angiogenesis

The hallmark of CCMs is the formation of fragile, hyperpermeable vascular lesions, driven fundamentally by excessive and dysregulated angiogenesis. This process is initiated by genetic insults but executed and amplified by a cascade of downstream signaling perturbations. The genetic foundation, as detailed in above sections, involves biallelic LOF in *CCM1*, *CCM2*, or *CCM3* genes, fitting a classic “two-hit” paradigm. Crucially, this genetic loss destabilizes endothelial quiescence, priming cells for a pro-angiogenic switch. Emerging evidence suggests a more complicated “multi-hit” landscape, where somatic mutations in genes such as *PIK3CA* or *MAP3K3* cooperate with LOF of CCM genes to accelerate lesion progression and modulate phenotype severity [25, 26, 83]. *PIK3CA* GOF enhances EC proliferation to exacerbate vascular malformation [5], yet its < 20% allele frequency in human CCMs questions its universal relevance. While *PIK3CA*-*CCM1* LOF synergism is validated in mouse models, species-specific vascular differences obscure translational applicability. MEKK3, which interacts with CCM2, is critical for embryonic angiogenesis [84–86]. The *MAP3K3 c.1323C* > G (p.Ile441Met) mutation is prevalent in mulberry-like lesions [26], but whether this drives other subtypes or is a mere marker remains controversial. Notably, *MAP3K3* GOF alone suffices to induce CCM-like angiogenic lesions (unlike *PIK3CA* GOF, which only aggravates growth) [26], a distinction untested in aged models mimicking adult-onset disease. A recent study reported that *MAP3K3*^I441M^ and *PIK3CA*^H1047R^ double mutations synergistically amplified PI3K-AKT-mTOR signaling, inducing an “angiogenic switch” reminiscent of tumor neovascularization [87]. Endogenous regulators further shape the angiogenic imbalance in CCMs. KLF2/4 activation acts as a master transcriptional regulator, driving a pro-angiogenic program. A primary downstream effect of KLF2/4 is the suppression of Thrombospondin-1 (*THBS1*), a potent endogenous angiogenesis inhibitor [64, 88, 89]. The loss of TSP-1 represents a critical permissive step, removing a key checkpoint that normally restrains endothelial proliferation and migration [64]. This mechanism is strongly supported by the phenocopy of CCM-like dilated vessels in TSP1-null mice [90, 91]. Simultaneously, VEGF signaling is hyperactivated (3-fold higher VEGF, 2-fold lower VEGFR1) via CCM3-mediated VEGFR2 modulation [92, 93], though the relative contribution of astrocyte or stromal cell-derived VEGF remains debated due to lack of direct comparative studies [94]. The Angiopoietin-TIE2 system is perturbed, with CCM3 LOF leading to increased secretion of antagonistic ANGPT2, disrupting vascular stabilization signals from ANGPT1/TIE2 [95, 96]. Similarly, the β1 integrin-KLF2-EGFL7 axis is dysregulated, leading to overexpression of the pro-angiogenic factor EGFL7 [65].

**Fig. 3:**
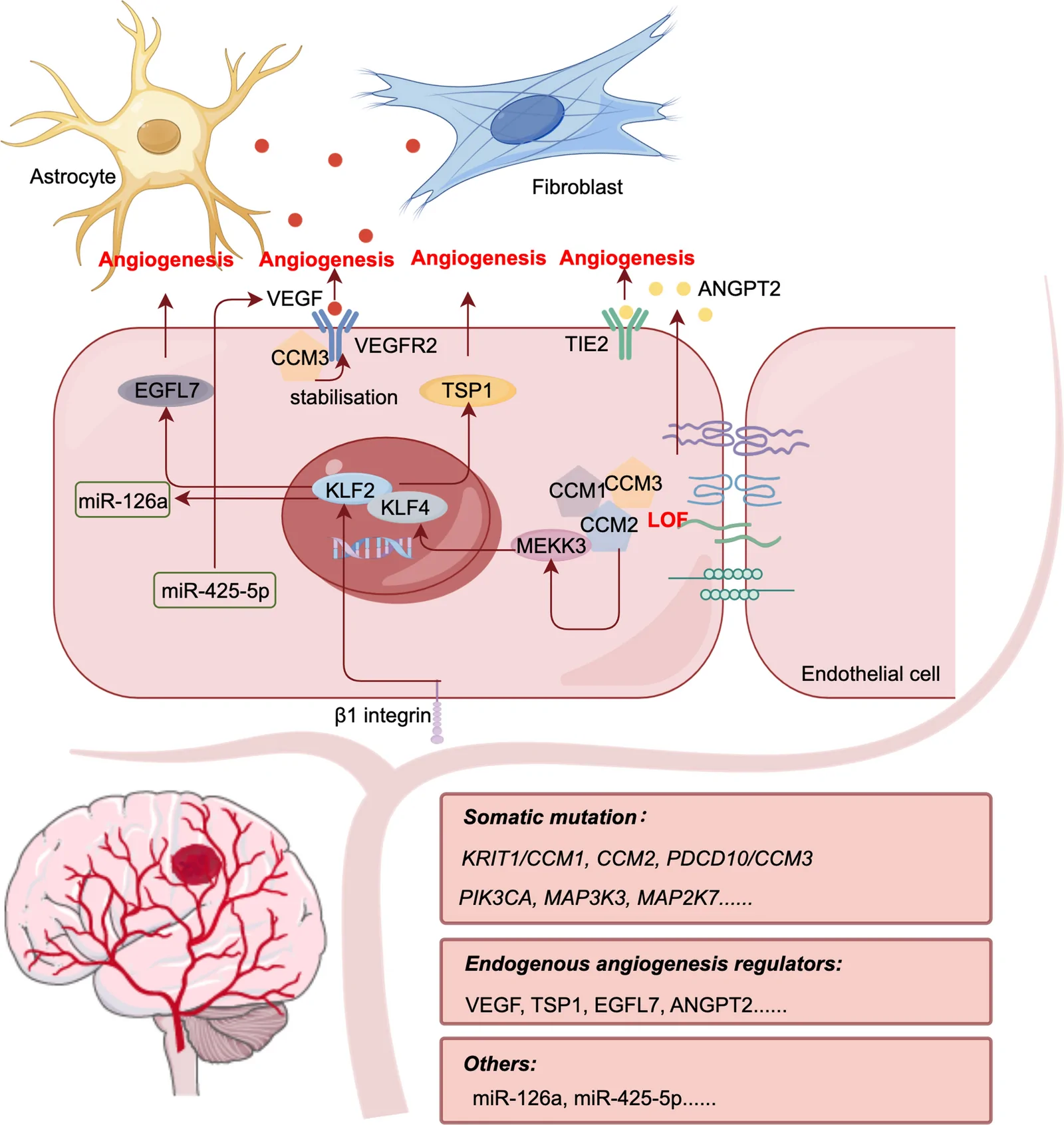
Excessive angiogenesis in CCMs is governed by multiple components. CCM1, CCM2, and CCM3 that are closely associated with development of CCMs normally function to repress angiogenesis and maintain endothelial quiescence and integrity. Additionally, other genes including *PIK3CA*, *MAP3K3*, and *MAP2K7*, when mutated, result in excessive angiogenesis by virtue of a triple-hit model. The interactions between the CCM genes and alternative genes like *PIK3CA* and *MAP3K3* promote the proliferation of ECs and lesion growth. Endogenous angiogenesis modulators including VEGF, TSP1, ANGPT2 and EGFL7 also play crucial roles. VEGF signaling is hyperactivated in CCMs, increased VEGF released by astrocytes and fibroblasts contribute to angiogenesis. The level of TSP1 is suppressed, thereby removing a critical angiogenic checkpoint, but the secretion of ANGPT2 is elevated with disrupted endothelial integrity. The figure highlights the complicated interactions between genetic mutations and various signaling pathways leading to the excessive angiogenesis of CCMs. Abbreviations: VEGF, vascular endothelial growth factor; TSP1, thrombospondin‑1; ANGPT2, angiopoietin‑2; EGFL7, EGF‑like domain‑containing protein 7; *PIK3CA*, phosphatidylinositol‑4,5‑bisphosphate 3‑kinase catalytic subunit alpha; *MAP3K3*, mitogen‑activated protein kinase kinase kinase 3; *MAP2K7*, mitogen‑activated protein kinase kinase 7; VEGFR2, vascular endothelial growth factor receptor 2; KLF2, Krüppel‑like factor 2; KLF4, Krüppel‑like factor 4; TIE2, tyrosine‑protein kinase receptor 2

MicroRNAs are implicated to play pivotal roles in excessive angiogenesis of CCMs, as exemplified by endothelial-specific miR-126a that accelerates VEGF-mediated angiogenesis as downstream of KLF2 [97], miR-425-5p that promotes angiogenesis by activating p38/VEGF signaling and suppressing Notch signaling via downregulating CCM3 [98], as well as other uncharacterized microRNAs whose downregulation in CCMs may further disrupt angiogenic balance. Local inflammatory cues may also precipitate the augmented proliferation and angiogenesis observed in CCM lesions [99]. In summary, excessive angiogenesis in CCMs results from genetic loss of endothelial constraints, culminating in a coordinated signaling catastrophe: removal of the TSP-1 brake, augmentation of the VEGF and EGFL7 accelerators, and impairment of TIE2-mediated stabilization (Fig. 3).

#### Disrupted EC junctions to induce vascular leakage

EC junctions, including adherens junctions (AJs), TJs, and gap junctions (GJs) are pivotal for maintaining vascular structural integrity and barrier function [100–102], and their disruption leading to increased vascular leakage is a hallmark of CCMs [103–106]. TJs (comprising claudins, occludin, and zonula occludens [ZOs]) regulate brain endothelial tightness, while AJs (e.g., VE-cadherin) mediate adhesion and cytoskeletal remodeling [107]. LOF of CCM genes drives EC junction disorganization both in vitro and in vivo. A consistent phenotype in CCM lesions is impaired EC junction integrity, though critical inconsistencies exist. While most studies reported reduced immunoreactivity of TJ proteins (Claudin-5, ZO-1) and AJ components at EC contacts in CCM lesions [81, 96], others documented elevated Occludin, VE-cadherin, and CD31 in some human CCM tissues [108], highlighting patient-specific variability or uncharacterized modulators that may confound junction protein expression profiles.

**Fig. 4:**
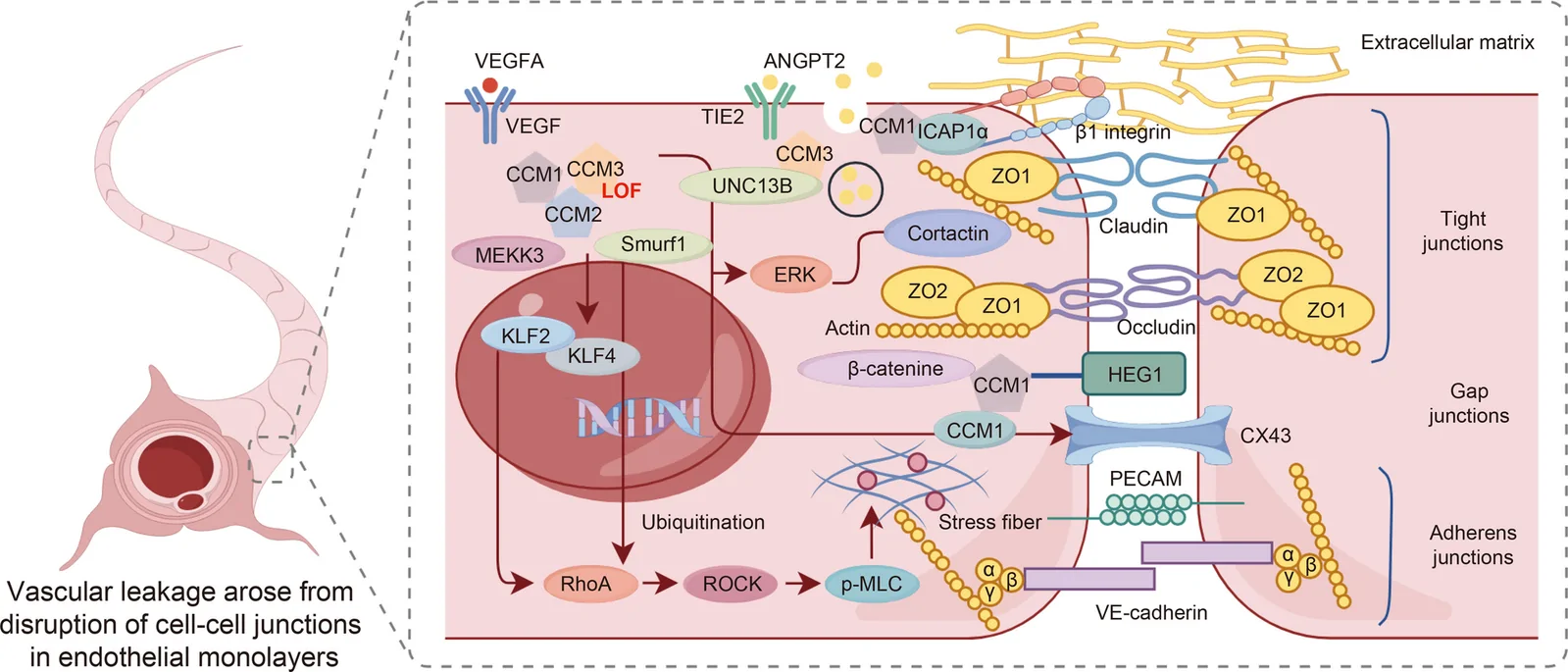
Underlying mechanisms of vascular leak in CCMs. CCM lesions are characterized by boosted vascular permeability owing to the destabilization of AJs, TJs, and GJs. LOF of CCM genes contributes to diminished expression and assembly of TJ proteins such as Claudin-5, Occludin and ZO-1, in particular in early lesions, leading to TJ destabilization and barrier dysfunction. Furthermore, elevated intracellular tension mediated through the RhoA/ROCK signaling cascade augments stress fiber formation and endothelial contractility, thereby disrupting TJs and exacerbating vascular leakage. LOF of CCM2 inhibits Smurf1-mediated RhoA degradation, increasing the activity of ROCK. CCM3 LOF disrupts exocytosis via increasing ANGPT2 secretion and destabilizing TIE2, which destabilizes EC junctions. Moreover, CCM3 LOF enhances the expression of CX43, a GJ protein, which orchestrates the communication of GJs and is associated with the redistribution of TJ proteins to GJs. Additionally, β1 integrin, which mediates EC‑matrix adhesion, is dysregulated upon CCM protein loss. CCM1 forms a complex with ICAP1α, and this interaction modulates β1 integrin‑dependent endothelial contractility and fibronectin remodeling, contributing to vascular leak. CCM1 and CCM3 LOF also potentiate the production of VEGF, which interacts with VEGFR2 to harness EC junction disassembly. CCM1 binds HEG1 and acts as a Rap1 effector to stabilize endothelial junctions via influencing the afadin/β-catenin interactions. Collectively, these mechanisms result in elevated endothelial permeability, micro-hemorrhages, and hemosiderin deposition in CCMs, highlighting the pivotal role of EC junction integrity and associated signaling pathways in the pathogenesis of CCMs. Abbreviations: AJs, adherens junctions; TJs, tight junctions; GJs, gap junctions; ZO‑1, zonula occludens‑1; ZO‑2, zonula occludens‑2; RhoA, Ras homolog family member A; ROCK, Rho‑associated coiled‑coil containing protein kinase; Smurf1, Smad ubiquitin regulatory factor 1; CX43, connexin 43; HEG1, heart of glass 1; Rap1, Ras‑proximate‑1; ICAP1α, integrin cytoplasmic domain‑associated protein‑1α; p‑MLC, phosphorylated myosin light chain; ERK1/2, extracellular signal‑regulated kinase 1/2; UNC13B, unc‑13 homolog B; PECAM‑1, platelet and endothelial cell adhesion molecule‑1

Conserved across human and animal models, RhoA/ROCK pathway activation in CCM-mutant ECs enhances intracellular tension, stress fiber formation, and myosin contraction, thereby disrupting TJs and exacerbating BBB dysfunction and iron deposition [79, 109, 110]. CCM2 constrains this pathway via Smurf1-mediated RhoA degradation [78, 111], while CCM1/2 complexes regulate ROCK1/2 balance [112]. Inhibition of ROCK mitigates CCM lesion burden in preclinical models, supporting downstream therapeutic potential though translatability to humans requires further validation. Notably, CCM3 deficiency uniquely alters both TJs (via CCM3-ERK1/2-cortactin signaling) [82] and GJs (via connexin 43 upregulation) [113]. Effects less prominent in CCM1/2-knockout cells, suggesting subtype-specific mechanisms that remain incompletely resolved. Furthermore, the interplay between CCM proteins and other signaling axes adds complexity. CCM3 LOF drives ANGPT2 secretion (via STK25-Unc13b) [96] and TIE2 dysregulation (via Cav1-caveolae) [95]. The CCM1-HEG1 complex stabilizes junctions independently of MEKK3-KLF2/4 signaling [114]. CCM1 as a Rap1 effector is secreted from microtubules and predominantly locates at cell–cell contacts, where it communicates with various components of EC junctions, such as afadin and β-catenin, to improve the stabilization of cell–cell junctions and further to limit permeability of EC monolayer [19, 115]. VEGF is accepted as a key regulator that both strengthens EC proliferation and disrupts endothelial junctions. However, only CCM1/3 (not CCM2) deficiency augments VEGF production in ECs, indicating unappreciated subtype-specific differences in pro-leakage signaling [116, 117]. β1 integrin, which mediates EC-matrix adhesion, is also dysregulated upon CCM protein loss. CCM1 forms a complex with ICAP1α (integrin cytoplasmic domain-associated protein-1α), and this interaction modulates β1 integrin-dependent endothelial contractility and fibronectin remodeling [118–120] (Fig. 4).

The causative hierarchy between junctional disruption, chronic micro-hemorrhage, and lesion growth is also unclear, junction leakage may be both a driver and a consequence of the abnormal vascular microenvironment. In summary, while dysfunction in EC junctions is unequivocally central to CCM pathology, the field must reconcile disparate mechanistic studies and address whether targeting junctional integrity itself offers a viable therapeutic strategy across all CCM genotypes.

#### Endothelial-mesenchymal transition (EndMT)

EndMT is a complex biological process wherein ECs lose endothelial traits (e.g., cell adhesion and polarity) and gain mesenchymal/stem-cell-like phenotypes [121, 122]. Cumulative evidence indicates that ECs in CCM lesions exhibit a mixed endothelial-mesenchymal phenotype, consistent with ongoing EndMT. CCM lesions originate from clonal expansion of mesenchymal-like ECs with CCM protein mutations, followed by wild-type EC incorporation, and EndMT is increasingly recognized as a likely contributor to CCM initiation and progression, although its causal role remains incompletely defined [123–125]. Mechanistically, CCM protein depletion reduces endothelial junctions-related molecules (VE-cadherin, Claudin-5) and upregulates mesenchymal/stemness markers (ID1, FSP1, SCA1, KLF4) [126]. The TGFβ/BMP cascade (notably BMP6) is a major trigger of EndMT in CCMs [63, 126, 127]. Dejana’s group demonstrated that CCM1 LOF activates the MEKK3-MEK5-ERK5-MEF2 pathway, elevating the expression of KLF4 in ECs [63, 126]. KLF4 then enhances TGFβ/BMP signaling via BMP6 induction, thereby promoting β-catenin nuclear translocation and ultimately regulating EndMT markers [63, 126–128]. KLF4 deletion attenuates EndMT in CCM models, though its role is cell-context-dependent. CCM1 knockout upregulates KLF4 in multiple EC types, but EndMT markers are only increased in brain ECs (BECs) [63], a limitation that may complicate translational relevance.

Additional regulators include miR-27b (enhancing TGFβ-induced EndMT) [129] and BMI1 (reversing upregulated KLF4/S100a4 in *Pdcd10*-null BECs) [130], suggesting potential therapeutic targets. However, inconsistencies do exist: Kahn’s lab found no early EndMT signaling alterations in CCM lesions, and KLF2 depletion retards CCM formation [61]. Moreover, CBX7/Cbx7a modulates KLF2-targeted EndMT genes, and *SPI1* was identified as a novel EndMT driver via scRNA-seq [131, 132]. These discrepancies are likely attributed to EndMT’s dynamic, time-dependent nature, a critical consideration often overlooked in cross-study comparisons. In summary, although mainstream evidence links EndMT to impaired endothelial barrier integrity and abnormal vascular lumen formation in CCMs, contextual variability in KLF4 function and conflicting temporal data highlight unresolved questions. Further studies addressing cell-type specificity and longitudinal EndMT dynamics are imperative to reconcile discrepancies and validate EndMT as a viable therapeutic target for CCMs. Whether EndMT is a driver of initiation or a mature-lesion state remains unresolved, with discrepant findings likely reflecting the time-dependent nature of this process.

#### Defective autophagy

**Fig. 5:**
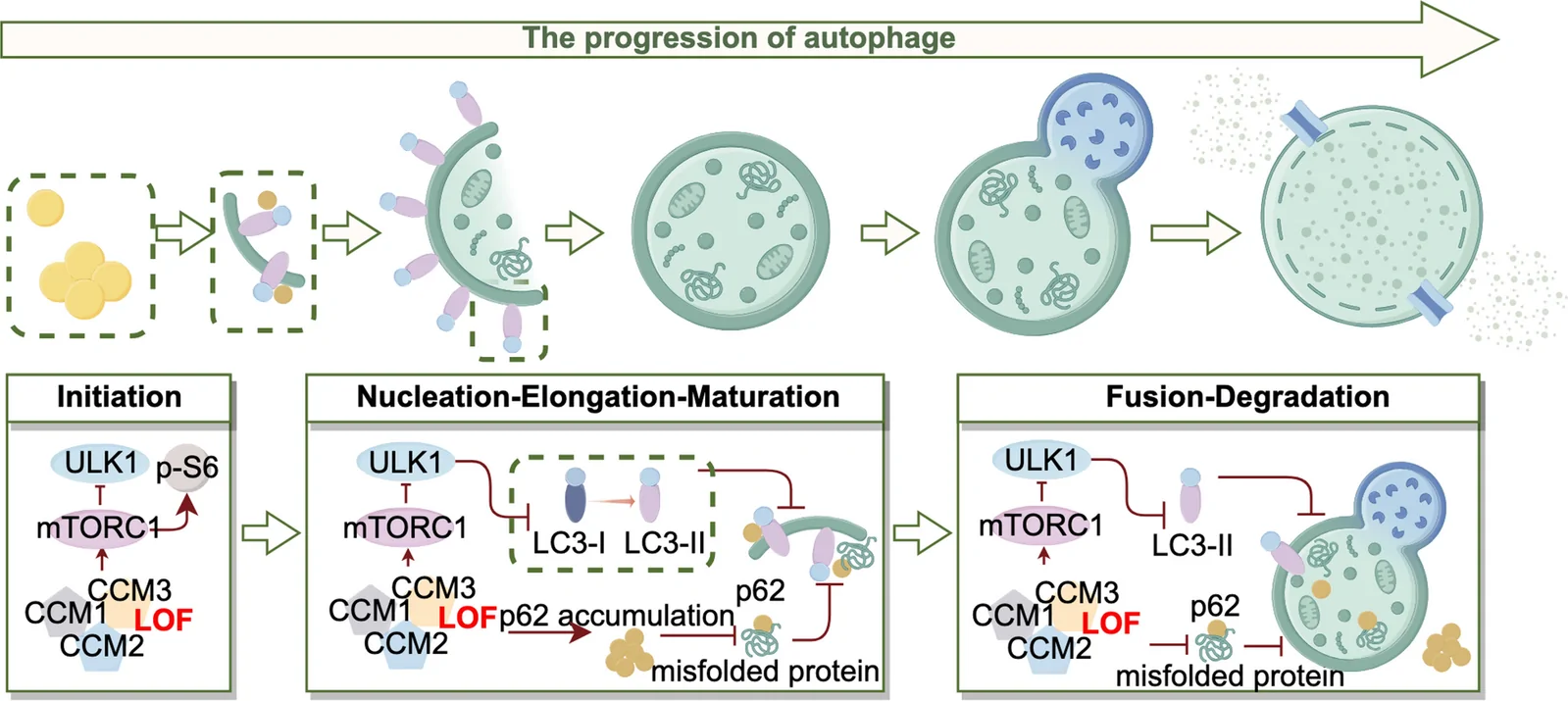
Defective autophagy in CCMs. The structural abnormalities of the CCM vessel wall result in the formation of “blood sinusoids”, where autophagy may emerge as a “cleaner”, and defective autophagy may exacerbate the damage of vascular ECs. The process of autophagy in CCMs and its related molecular mechanisms are described below. Autophagy initiation blockade: CCM genes LOF activates the mTOR signaling pathway, suppressing ULK1 activity and thus blocking autophagy initiation. p-S6 as a marker of mTOR activity is upregulated, indicative of mTOR-induced repression of autophagy. Defective autophagosome formation: inhibited ULK1 fails to promote LC3-I lipidation into LC3-II, reducing autophagosome membrane labeling (LC3-II puncta). LOF of CCM genes results in intracellular accumulation of p62, forming aggregates and yielding substrate clearance impairment. Fusion-lysosomal dysfunction: under normal conditions, p62 binds to its substrate and subsequently interacts with LC3-II, which facilitates the delivery of the substrate to lysosomes via autophagosomes for degradation. In CCMs, undegraded p62 accumulates, forming toxic aggregates that exacerbate stress and death of ECs. Abbreviations: mTOR, mammalian target of rapamycin; ULK1, Unc‑51‑like kinase 1; p‑S6, phosphorylated ribosomal protein S6; LC3‑I, microtubule‑associated protein 1 light chain 3‑I; LC3‑II, microtubule‑associated protein 1 light chain 3‑II; p62, sequestosome‑1 (SQSTM1)

Defective autophagy, a critical degradation process for maintaining cellular homeostasis, is increasingly recognized as an important element in the pathogenesis of CCMs [72, 133, 134]. Across all three CCM proteins, the phenotypes are uniform: sustained mTORC1 activation, ULK1 inhibition, p62/SQSTM1 aggregation and LC3-I accumulation [25, 71, 72, 135]. LOF of CCM genes consistently leads to impaired autophagic flux in ECs, primarily through hyperactivation of the PI3K-mTOR signaling pathway. mTOR inhibitors (Torin 1, rapamycin) are able to restore autophagy and rescue CCM-related phenotypes in CCM1-knockout cells, whereas mTOR-independent pro-autophagic agents (xestospongin B) fail [72, 136], highlighting the dominant role of mTOR in the pathways examined, although this reliance may not be universal across all CCM genotypes and experimental contexts. Vitamin D deficiency increases stroke risk [137, 138], and vitamin D mitigates CCM-related autophagy defects via mTORC1 inhibition [76], yet clinical translation is hampered due to limited direct evidence in CCM patients. Similarly, mitophagy has been invoked because CCM3 LOF fragments mitochondria and raises ATPIF1 [139], but whether mitophagy is blocked or excessively triggered remains contentious, highlighting the requirement for standardized mitophagy metrics. Nevertheless, is defective autophagy the only phenotype that accompanies to the occurrence of CCMs or can insufficient autophagy affect other pathological signatures that result in the progression of CCMs? It has been revealed that autophagy acts as a crucial biological behavior for curbing cell homeostasis [140], as well as plays pivotal roles in influencing EndMT and reactive oxygen species (ROS) production, which are accepted as two critical phenotypic profiles of CCMs [72, 134] (Fig. 5). Defective autophagy is primarily a downstream consequence of mTORC1 hyperactivation, as mTOR inhibitors restore flux whereas mTOR-independent agents fail to do so [72, 136]. Collectively, mTOR-driven autophagy inhibition is a core event in CCM pathogenesis. Defective autophagy directly promotes oxidative stress through impaired clearance of damaged mitochondria and facilitates EndMT by stabilizing pro-fibrotic signaling intermediates [56, 134]. However, gaps persist including subtype-specific pathway differences and unconfirmed clinical relevance of preclinical findings, warranting further research to resolve ambiguities.

#### Inflammation and immune response

Substantial evidence underscores inflammation as a pivotal driver in the pathogenesis of CCMs, which serves as both a primary amplifier of lesion progression and a secondary response to microhemorrhage and iron deposition [141–143]. CCM lesions are infiltrated by diverse immune cells, including monocytes, macrophages, and B and T cells, which foster a chronic inflammatory milieu that propels disease progression and clinical severity. Transcriptomic and proteomic analyses consistently reveal ECs as central orchestrators, upregulating a suite of cytokines and chemokines (e.g., CCL2, CXCL1, IL-1β) upon loss of CCM genes, thereby recruiting and activating immune populations [142, 144, 145]. Key inflammatory mechanisms have been delineated. Neutrophils are rapidly recruited, forming neutrophil extracellular traps (NETs) that aggravate immunothrombosis [142, 146, 147]. Pharmacological blockade of NETs attenuates immunothrombosis and neuroinflammation in CCMs [148]. Depletion of B cells halts CCM progression in mice, highlighting their functional roles [149]. The MEKK3-KLF2/4 signaling cascade, crucial for CCM complex formation, integrates inflammatory stimuli, in particular TLR4 activation by LPS, with hemodynamic forces [66, 68, 150]. Polymorphisms enhancing TLR4/CD14 expression correlate with increased lesion burden in CCM patients [66]. Furthermore, vitamin D deficiency, which is associated with low-grade inflammation, emerges as a modifiable risk factor. Supplementation of vitamin D shows protective effects in models by dampening pro-inflammatory pathways [75, 77, 151].

**Fig. 6:**
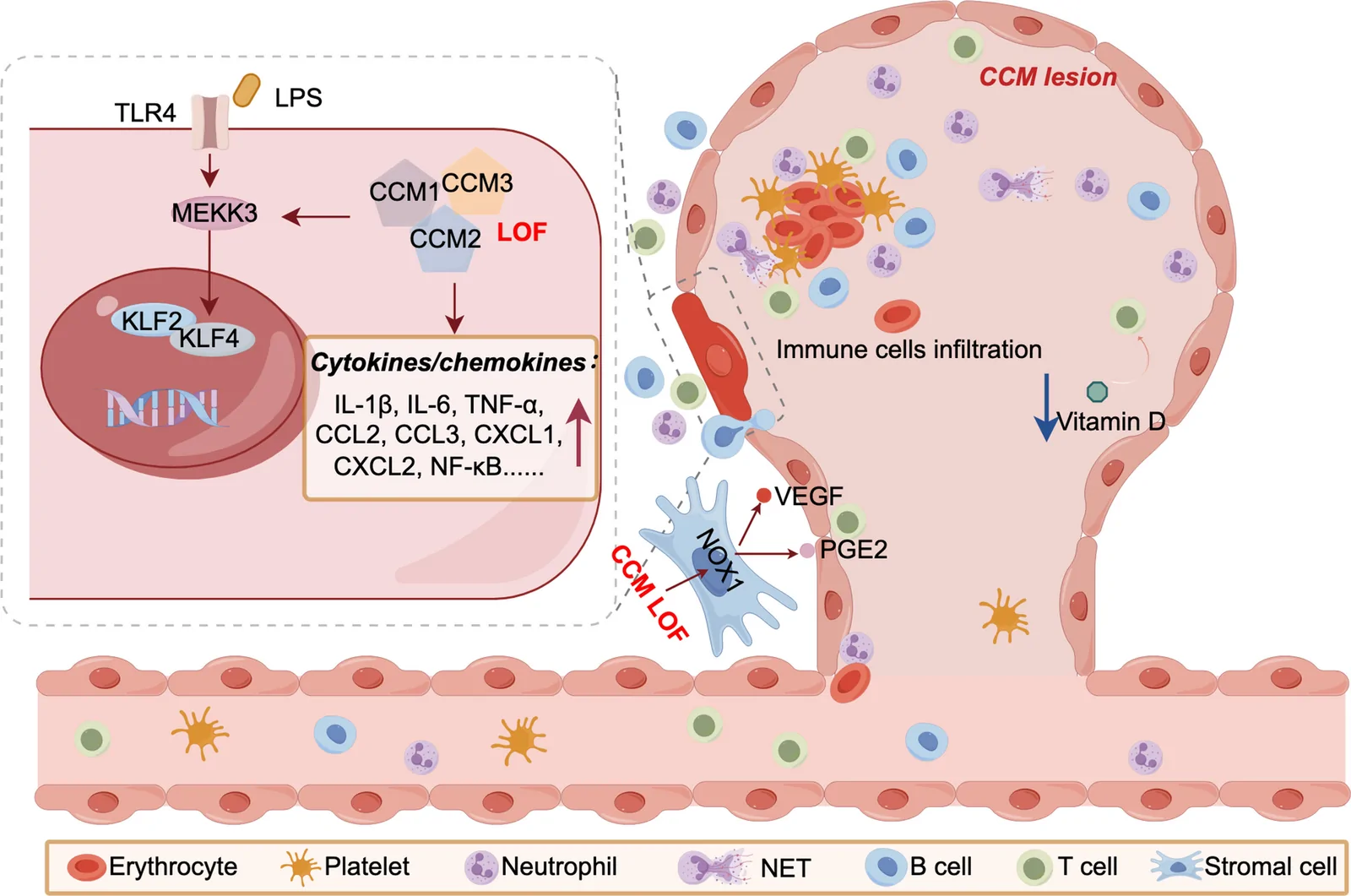
Inflammation and immune response in CCMs. This figure elucidates the important role of inflammation and immune response in the initiation and progression of CCMs. Critical events include the infiltration of immune cells (e.g., neutrophils, macrophages, B cells, and T cells) into CCM lesions, contributing to chronic inflammation and release of various pro-inflammatory cytokines and chemokines (e.g., IL-1β, IL-6, TNF-α, CCL2, CXCL1). Immune cells such as neutrophils result in immunothrombosis via NETs. Additionally, an array of signaling pathways including MEKK3-KLF2/4, TLR4, NF-κB, and JNK/c-Jun/COX-2 signaling cascades are activated, integrating inflammatory and hemodynamic signals. Genetic and environmental factors, such as LOF mutations in CCM proteins, vitamin D deficiency, and polymorphisms in inflammation-associated genes, further aggravate disease severity. These mechanisms collectively drive CCM lesion growth, hemorrhage, and clinical symptoms such as seizures. The figure highlights the associations between inflammation, immune cell infiltration, and molecular signaling cascades in the progression of CCMs, providing insights into potential therapeutic targets. Abbreviations: IL‑1β, interleukin‑1β; IL‑6, interleukin‑6; TNF‑α, tumor necrosis factor‑α; CCL2, C‑C motif chemokine ligand 2; CCL3, C‑C motif chemokine ligand 3; TLR4, toll‑like receptor 4; NF‑κB, nuclear factor‑κB; JNK, c‑Jun N‑terminal kinase; COX‑2, cyclooxygenase‑2; NETs, neutrophil extracellular traps; LPS, lipopolysaccharide; CXCL1, C‑X‑C motif chemokine ligand 1; CXCL2, C‑X‑C motif chemokine ligand 2

However, a critical assessment reveals notable controversies and gaps. While inflammation is broadly implicated, its specific contributions to different clinical phenotypes, such as epilepsy versus symptomatic hemorrhage, are only beginning to be dissected. Recent evidence suggests inflammatory enzyme activity (e.g., COX-2, NLRP3) may be more pronounced in hemorrhagic presentations compared to epileptic ones, hinting at divergent pathways [152]. The correlation between specific cytokine levels and hemorrhage remains conflicted, particularly for IL-6, with studies reporting both positive and negative associations [153, 154]. This inconsistency underscores the complexity of translating plasma biomarker findings into reliable clinical predictors (Fig. 6). In conclusion, inflammation is increasingly recognized as an important contributor in CCMs, offering a promising therapeutic target. Nonetheless, the field must move beyond mere association to define causal, phenotype-specific inflammatory circuits. Future studies should prioritize resolving biomarker discrepancies, validating targets in human tissues, and designing clinical trials that account for the heterogeneity of CCM inflammatory signatures.

#### Redox homeostasis imbalance and oxidative stress

CCM proteins are critically involved in cellular defense against oxidative stress. LOF mutations in CCM genes, particularly CCM1, disrupt redox homeostasis in ECs. Major research findings regarding the association between CCMs and oxidative stress have been published by Retta’s group, who demonstrated that CCM1 LOF elevates intracellular ROS, impairs mitochondrial metabolism, diminishes FoxO1-SOD2-dependent antioxidant signaling, and increases susceptibility to oxidative damage [155, 156]. The group further clarified that these pathways fail to act in isolation, they identified Nd1-L as a novel molecular interactor of CCM1, showing that this interaction modulates SOD2 expression, and also found that CCM1 LOF disrupts the balance between pro-oxidant (c-Jun/COX-2) and adaptive antioxidant (Nrf2/HO-1/GLO1) axes [157–159]. In addition, CCM1 LOF disrupts the glutathione system, reducing GSH/GSSG ratios and inducing widespread protein S-glutathionylation modifications that affect chaperones, metabolic enzymes, and cytoskeletal regulators, thereby implying a distinctive redox proteomic signature in CCM1-deficient cells [160].

In human studies, polymorphisms in oxidative stress‑related genes, including CYP and MMP family members, correlate with CCM1 disease severity and ICH risk, supporting interindividual variability in oxidative stress susceptibility as a disease regulator [18, 49]. Nevertheless, conflicting evidence highlights that excessive antioxidant signaling can induce reductive stress, challenging the assumption that ROS reduction is universally beneficial [161, 162]. This hormetic response complicates the design of antioxidant therapies and warrants cautious interpretation of preclinical antioxidant trials. The field must also confront the possibility that oxidative stress, while contributory, may be a downstream consequence rather than a primary driver, intertwines with inflammatory pathways (oxy-inflammation) and autophagic dysfunction. Oxidative stress is best understood as a dynamic redox imbalance, excessive antioxidant signaling can induce reductive stress, suggesting homeostasis restoration as the therapeutic goal. Ultimately, although targeting redox imbalance is a compelling therapeutic avenue, a deeper understanding of the context-dependent and often adaptive nature of these responses is essential for moving beyond the current antioxidant paradox.

#### Influenced gut microbiome

A growing body of evidence, derived largely from preclinical models, has established a mechanistic link between the gut microbiome and the pathogenesis of CCMs. This link was first suggested by the observation that neonatal mice subjected to the same induction of CCM disease displayed strikingly divergent CCM burdens when they were housed in different animal facilities [66]. This led to the identification of LPS from gram-negative bacteria (GNB) as a key driver. LPS activates Toll-like receptor 4 (TLR4) on brain ECs (BECs), triggering the MEKK3-KLF2/4 signaling cascade that is central to CCM formation [46, 66]. Consistently, antibiotic treatment ameliorates CCM development in mouse models, while administration of LPS exacerbates it [22, 66]. The gut-brain axis in CCMs is bidirectional. Beyond the well-established roles of CCM complexes in vascular endothelium, CCM3 (PDCD10) plays a critical gatekeeper function in the gut. It is required for mucus production by intestinal goblet cells, and its heterozygous loss compromises the gut barrier, leading to increased LPS translocation into the circulation and heightened brain lesion burden [5, 21, 22]. This dual role suppressing CCM in the brain and maintaining barrier integrity in the gut helps explain why CCM3 LOF often lead to the most severe clinical phenotypes [5]. In line with this, human cohort studies show that CCM patients harbor a distinct gut microbiome with an enrichment of LPS biosynthesis genes compared to healthy individuals, directly correlating microbial composition with CCM disease progression [163]. However, these human data are observational, and the causal contribution of gut-barrier dysfunction to human CCM severity, as well as any therapeutic implication of microbiome modulation, remains to be proven.

However, the relationship is not solely microbe-dependent. The dietary emulsifier polysorbate − 80 (P80) can promote CCM formation in mice by damaging the mucus barrier without altering the microbiome composition, suggesting that barrier integrity itself is an independent variable [22]. Furthermore, CCM1 has been implicated in intestinal epithelial barrier function and response to pathogens [164, 165], and its loss can enhance β-catenin signaling, potentially linking CCM mutations to broader gastrointestinal pathophysiology [166, 167]. These findings present a nuanced view, while GNB-derived LPS is a potent trigger, the physical integrity of the gut barrier acts as a critical modulator that can influence CCM severity independently. This complexity presents exciting therapeutic avenues, including dietary intervention, fecal microbiota transplantation, or barrier fortification, though precisely disentangling the interdependent effects of microbial composition and barrier function remains a significant challenge for translating these insights into targeted clinical strategies.

#### Altered brain vascular microenvironment

The pathogenesis of CCMs extends beyond mere homozygous loss of CCM genes in ECs, as evidenced by the spatial and temporal exclusivity of lesions in animal models despite pan-endothelial gene deletion [12, 61, 168, 169]. This foundational observation necessitates a critical examination of the brain vascular microenvironment as the critical modifier that dictates where and when lesions form. Clinical heterogeneity, even among family members with identical mutations [48, 170], further underscores that genetic predisposition is necessary but insufficient, requiring local “hits” from microenvironmental stressors to initiate and exacerbate the disease.

Hemodynamics or fluid shear stress (FSS) is arguably the best-defined local trigger. The predilection of CCM lesions for low-FSS regions, such as the brain and retina [67, 126, 171], is not merely correlative. Low FSS robustly activates the MEKK3-MEK5-ERK5-KLF2/4 signaling axis [67, 172], a pathway normally involved in cardiovascular development but pathologically sustained in CCM gene deficient ECs. This mechanotransduction event results in the upregulation of downstream effectors like ADAMTS4/5, which remodel the extracellular matrix [69]. While the paradigm that low FSS is pathogenic is compelling, a critical caveat is the inherent difficulty in isolating FSS from other concurrent flow-related phenomena (e.g., oxygen tension, nutrient delivery). Furthermore, clinical observations that elevated systemic blood pressure correlates with fewer lesions [57] seemingly contradict the low-FSS model, emphasizing that systemic hemodynamic factors such as blood pressure must be distinguished from the local wall shear stress acting on lesional endothelium. The field must therefore reconcile how systemic pressure increases can be protective while local low flow is detrimental, possibly through differential effects on established versus nascent lesions. The role of FSS is also intimately correlated to cell mechanics, where loss of CCM proteins leads to hyperactivation of β1 integrin and RhoA, thereby increasing EC contractility and predisposing vessels to rupture in these low-flow zones [118, 171, 173, 174]. Low FSS in brain venous capillaries, combined with *PIK3CA* GOF mutations as a “third hit” exacerbates CCM lesion progression by hyperactivating PI3K/AKT/mTOR signaling and disrupting endothelial quiescence [175].

BBB and its constituent neurovascular unit (NVU) represent another critical layer of microenvironmental impacts. CCM lesions are characterized by a profound loss of normal NVU architecture, including detachment of pericytes and astrocytic end-feet [176, 177]. This is not a passive consequence but an active, bidirectional process. EC-specific loss of CCM3, for instance, disrupts the Angiopoietin-Tie2 axis, weakening EC-pericyte adhesion [178]. Conversely, pericyte-specific CCM3 loss impairs their migration and recruitment, demonstrating a non-cell-autonomous role for CCM proteins in mural cells [179]. A key controversy, however, lies in the temporal sequence: is pericyte loss a primary trigger for lesion formation or a secondary effect of a dysfunctional endothelium? Evidence for both exists [176, 178, 179], suggesting a vicious cycle that is difficult to disentangle. Similarly, astrocytes are not innocent bystanders. Their activation and proliferation in CCM models [180, 181] create a feedback loop where EC-derived nitric oxide stabilizes HIF-1α in astrocytes, prompting VEGF secretion that further destabilizes the vasculature [94]. This interplay highlights that CCM is a disease of the entire NVU, yet most therapeutic strategies remain EC-centric, a point of potential translational limitation. The role of microglia, while increasingly recognized [143, 182–184], remains the most nebulous, whether their presence drives lesion progression or represents a reactive cleanup effort is still debated and requires functional studies beyond observational histology. scRNA-seq has further illustrated the cellular heterogeneity within the NVU, thereby reinforcing a maladaptive neurovascular crosstalk [185].

Finally, the dynamic and heterogeneous nature of CCM lesions is starkly demonstrated by the behaviors of blood components. The classic view of CCMs as purely hemorrhagic lesions is an oversimplification. The presence of both thrombi and hemorrhage within the same or different lesions [186–188] points to a highly unstable and spatially heterogeneous endothelium. This is mirrored by the variable expression of thrombomodulin [187, 189, 190] and the accumulation of von Willebrand factor (vWF) [191], which can simultaneously create pro-coagulant and anti-coagulant microenvironments and reflect an unstable, barrier-dysfunctional endothelium [192]. The finding of polyhedrocytes in fCCM inversely correlating with thrombomodulin expression [191] suggests a complicated crosstalk where the clot itself may alter the endothelial phenotypes. A critical appraisal of this area must acknowledge that our understanding of the coagulation cascade in CCMs is largely inferred from static snapshots (surgical resections) or in vitro models. The precise trigger that shifts an EC from a vWF-secreting, pro-thrombotic state to a bleeding-prone phenotype in vivo remains unknown, and the clinical significance of stable clot formation versus recurrent micro-hemorrhages in driving CCM lesion growth is a crucial unanswered question.

**Fig. 7:**
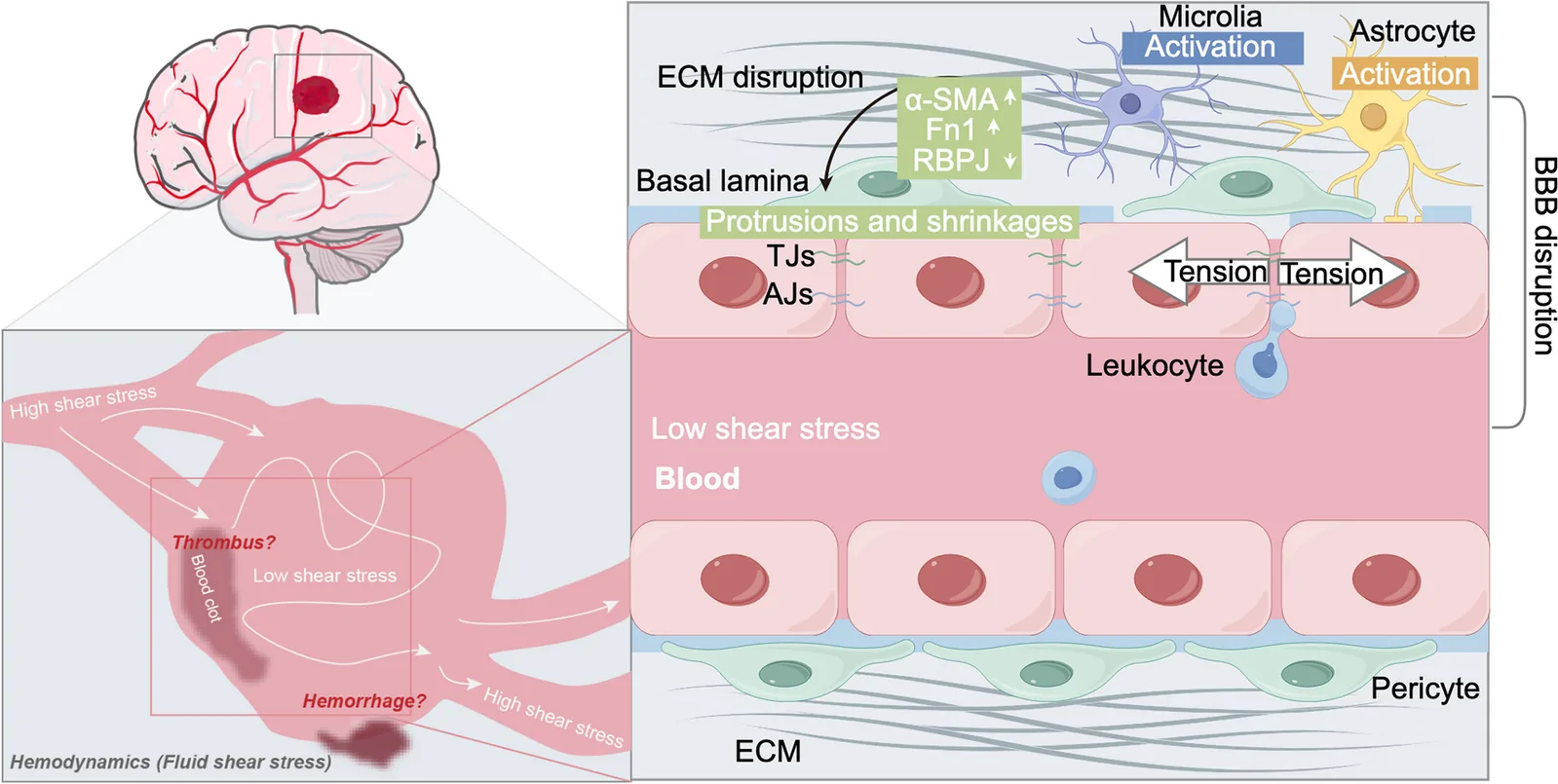
Mechanistic overview of roles of brain vascular microenvironment in CCMs. This figure provides a comprehensive overview of hemodynamic forces, BBB dysfunction, ECM remodeling, and hemostatic dysregulation in the pathogenesis of CCMs. Low FSS, predominant in areas of impaired blood flow, activates pivotal signaling pathways to yield endothelial dysfunction, vascular instability, and hemorrhagic stroke risk. Likewise, structural and functional disruptions within NVU, including pericyte loss, astrocyte activation, and endothelial junction disruption, tend to propel CCM lesion formation and progression. These alterations are further aggravated by dysregulated cell–matrix interactions, where CCM proteins modulate integrin-mediated adhesion, leading to ECM stiffness, cytoskeletal reorganization, and overexpression of matrix-remodeling mediators. In addition, dynamic hemostatic environment within CCM lesions, in particular thrombosis-prone regions and hemorrhage-prone areas, is characterized by vascular heterogeneity and dysfunction. Dysfunctional ECs are inclined to induce intrinsic and extrinsic coagulation cascades to recruit platelets and inflammatory cells, thereby destabilizing lesions and provoking their growth and clinical heterogeneity. Interactions between ECs, pericytes, astrocytes, and immune cells, coupled with biomechanical and biochemical stressors, collectively drive lesion initiation, growth, and clinical variability of CCMs. Abbreviations: FSS, fluid shear stress; BBB, blood‑brain barrier; ECM, extracellular matrix; NVU, neurovascular unit; ECs, endothelial cells; Fn1, fibronectin 1; RBPJ, recombination signal binding protein for immunoglobulin kappa J region; α‑SMA, alpha‑smooth muscle actin

In summary, the CCM lesion is a product of its microenvironment (Fig. 7). Genetic predisposition creates a vulnerable endothelium, but it is the confluence of disturbed blood flow, dysfunctional cellular interactions within the NVU, and a volatile hemostatic landscape that dictates CCM disease progression. Furthermore, patient-derived BBB assembloids generated from human pluripotent stem cells have recently recapitulated key features of CCMs, including endothelial junction disruption and the developmental loss of vascular smooth muscle cells [193]. Recognizing this integrated, multi-factorial pathogenesis is essential for moving beyond single-target therapies and towards a more holistic approach to manage this complicated vascular disease.

## Diagnosis and clinical presentation

### Neuroradiological diagnosis and clinical presentation

The diagnosis of CCMs relies heavily on characteristic neuroradiological findings, with MRI serving as the gold standard. MRI is the preferred diagnostic modality in all non-emergent situations, achieving nearly 100% sensitivity and high specificity for the diagnosis, characterization, and longitudinal follow-up of CCMs [28, 34]. The typical and featured phenotypes of CCMs are best demonstrated on MRI, where specific sequences allow for detailed evaluation. When diagnosing CCMs via MRI, crucial aspects to consider include the size and location of the lesions, as well as evidence of dynamic changes. Key observations are composed of the presence of acute or recurrent intracranial hemorrhage, brain edema, an intracranial mass effect or brain herniation, growth of the lesions, alterations in the appearance of blood components, as well as the development of new CCMs over time [8, 34, 194, 195]. On the basis of the appearance of CCMs on MRI, four forms of CCM lesions (Type I–IV) have been well described. Type I lesions are subacute and T1 hyperintense, Type II lesions are characterized by mixed signals and represent classic “popcorn” CCM lesions, Type III lesions are thought to be chronic hypointense lesions, and Type IV lesions appear to be tiny lesions observed only when small hypointense foci on susceptibility sensitive sequences like gradient-echo or SWI [195, 196].

Modern diagnostic techniques have significantly enhanced detection accuracy. SWI, for instance, is more precise in identifying CCM lesions, particularly small Type IV lesions, compared to older modalities like T2-weighted gradient echo sequences [29, 184]. These advanced techniques have also demonstrated that new lesions can develop de novo with age. Quantitative susceptibility mapping (QSM), a gradient echo-generated methodology used to evaluate iron deposition, is another emerging tool that may prove to be useful for CCM diagnosis [197, 198]. While MRI is paramount, computed tomography (CT) remains a frequently utilized tool, especially in acute settings. CT is a relatively quick, widely available, and inexpensive technique that is very helpful for diagnosing hemorrhage, edema, and mass effect in patients presenting with acute symptoms [8, 194]. However, its sensitivity to small CCMs is low, and it can only provide suggestive, rather than specific, diagnostic conclusions [8]. Any suspicion raised on a CT scan should be confirmed with MRI. Of note, the radiation risk relevant to de novo CCM formation derives primarily from therapeutic cranial irradiation rather than from diagnostic-level CT exposures, nonetheless, minimizing avoidable repeated CT examinations remains prudent [199, 200].

Although numerous CCMs exhibit these characteristic imaging features, diagnosis can be challenging, as other hemorrhagic lesions may present with a similar appearance. The clinical presentation is highly variable. In fact, 50%−70% of CCM patients are asymptomatic, and their lesions are discovered incidentally due to the widespread use of MRI [201]. When symptomatic, the clinical images depend on the lesion’s location. While primarily a brain disorder, cavernous malformations can also occur in the spinal cord, where they may contribute to spinal cord hemorrhage, edema, and myelopathy [202, 203]. In such cases, brain imaging is often required to assess for concurrent CCMs and aid in diagnosis [204]. Machine learning models integrating clinical and imaging features can help predict the risk of (re)hemorrhage in patients with sCCM, potentially aiding personalized surveillance strategies [205].

### Molecular genetics and integrated diagnostic strategy

Complementing neuroradiology, genetic testing plays an indispensable role in the diagnosis of CCMs, in particular for patients with multiple lesions. A diagnosis of fCCM, which follows an autosomal dominant pattern of inheritance with deficient clinical and neuroradiological penetrance, is strongly suggested when numerous lesions are present [30]. In such patients, or those with a positive family history, genetic testing for mutation or deletion analysis of the three known CCM genes is highly recommended [9]. More than 95% of fCCM cases and 60% of sCCM cases presenting with multiple lesions harbor LOF mutations in one of the three classic CCM genes [36, 206]. These genes are mapped to three distinct loci, *CCM1* on chromosome 7q, *CCM2* on 7p, and *CCM3* on 3q, with the majority of fCCM cases linked to the CCM1 locus [1, 207]. Most mutations, regardless of the underlying mechanism (e.g., nonsense mutations, deletions, insertions, or intronic mutations leading to aberrant splicing), tend to create a premature stop codon, resulting in a truncated, non-functional protein [208].

To ensure robust diagnostic sensitivity, multiple methodologies are employed. Genetic testing is recommended for patients with multiple lesions, a positive family history, or early‑onset/recurrent hemorrhage. For families with an identified mutation, cascade screening of at‑risk relatives is advised. Pre‑ and post‑test genetic counseling is essential for addressing disease penetrance, surveillance strategies, and family planning [9]. This typically involves sequencing all coding exons and exon/intron boundaries for the three CCM genes from genomic DNA isolated from blood, often complemented by copy number analysis to detect large deletions or duplications [208, 209]. However, despite comprehensive screening, a diagnostic gap remains. Mutations are not detected in approximately 5% of familial cases and up to 40% of sporadic cases with multiple lesions [208, 210]. The identification of CCM gene loci is still clinically useful, as it enables the identification of mutation carriers among family members and facilitates the evaluation of disease penetrance. The integration of neuroimaging and genetic data enables personalized risk stratification in clinical practice. MRI with susceptibility-weighted sequences allows classification of lesions by type (I-IV), which provides prognostic information on hemorrhage risk [195]. SWI and QSM enable quantification of iron deposition, with higher susceptibility values reflecting prior hemorrhage and predicting future bleeding events [197]. Genetic testing stratifies patients by genotype, *CCM3* mutations are associated with earlier onset and more severe disease, whereas *CCM2* mutations tend to present with milder phenotypes [16]. The presence of multiple lesions or a positive family history should prompt genetic testing, which is also essential for identifying asymptomatic mutation carriers who may benefit from surveillance and for counseling family members. Integration of these imaging and genetic data enables risk-stratified management, including identification of high-risk patients requiring more frequent follow-up, selection of candidates for clinical trials, and personalized surgical planning.

In conclusion, the contemporary diagnostic approaches to CCMs have evolved into a synergistic model that integrates advanced neuroimaging with molecular genetics. MRI with susceptibility-weighted sequences remains the diagnostic cornerstone due to its near-perfect sensitivity and ability to characterize lesions. Genetic testing is essential for patients presenting with multiple lesions or a familial history, confirming the diagnosis and enabling family counseling. However, challenges persist, including the need to weigh the (predominantly therapeutic) radiation-related risk of de novo lesion formation against the judicious use of CT in acute settings, as well as unresolved diagnostic gap in mutation-negative cases. Moving forward, an integrated strategy that correlates dynamic imaging features with genomic data is essential, not merely for accurate diagnosis, but for personalized risk stratification and long-term surveillance.

## Therapeutic strategies

### Surgical management strategies for CCMs

Clinically nascent CCM lesions have a low initial hemorrhagic risk (< 0.5%), yet symptomatic hemorrhage leads to high recurrence likelihood and is the primary indication for intervention as the most life-threatening complication. CCM clinical management is guided by expert consensus and guidelines [8, 173], with decisions based on lesion morphology, location, symptom severity and prior hemorrhagic history. Surgical resection of accessible symptomatic lesions (e.g., those that have hemorrhaged or caused refractory epilepsy) is the most commonly used first-line strategy [8, 31, 211], whereas observation is preferred for asymptomatic lesions, and stereotactic radiosurgery (SRS) is reserved for selected surgically inaccessible lesions for which the evidence of hemorrhage prevention remains limited. Preoperative patient functional assessment is fundamental for surgical planning, but over two decades of neurosurgical experience has left the evidentiary basis for CCM excision controversial. Resection is the mainstay for high-risk patients but entails notable costs and non-negligible morbidity/mortality, especially for lesions in the brainstem and other eloquent regions [8]. Clinical studies with > 20 symptomatic patients have failed to confirm definitive surgical net benefit or harm, only limited findings support its efficacy for CCM-related epilepsy, with most studies compromised by inherent biases [212, 213].

Surgical risk varies drastically by lesion location, making rigorous comparison of surgical risk and CCM natural history critical for individualized decisions [214, 215]. Resection is generally recommended for symptomatic, easily accessible CCMs, given elevated post-first-hemorrhage rebleeding risk and relatively low procedural morbidity [195, 196]. Management strategies also differ across clinical contexts, pediatric CCMs require consideration of age‑dependent natural history and long‑term outcomes [45], brainstem lesions demand careful risk‑benefit assessment due to high surgical morbidity [216], and spinal CCMs often warrant resection when symptomatic, with intraoperative monitoring to minimize neurological injury [203]. Lesions involving visual pathways or the lateral ventricle require specialized consideration [217–219], and adjunctive technologies (image guidance, neurophysiological monitoring, laser-assisted techniques) are claimed to improve eloquent-region outcomes, despite a lack of controlled studies validating specific methodologies [213, 220–223]. SRS is viable for solitary, surgically inaccessible lesions or those with high procedural risk but is not recommended for asymptomatic lesions or fCCM [224], while laser interstitial thermal therapy is an emerging minimally invasive investigational approach [225]. Surgical planning requires integrated anatomical and neuroradiological evaluation to select candidates and reduce complications, with neuronavigation, intraoperative ultrasound and neurophysiologic monitoring routinely used for lesion localization. The Alliance to Cure Cavernous Malformation (https://www.alliancetocure.org/home/for-patients/newly-diagnosed/ccm-care-guidelines/) provides peer-reviewed clinical recommendations, synthesizing controversies and knowledge gaps [34], yet current surgical practice remains heavily reliant on retrospective data and expert opinion due to the absence of large prospective randomized controlled trials (RCTs), thereby possibly introducing selection bias and limiting generalizability.

### Pharmacological therapies for CCMs

Given surgical limitations for multiple, deep-seated, or eloquent-area lesions, pharmacological interventions represent an urgent unmet need [226]. While preclinical studies have identified numerous potential targets, clinical translation remains slow, with few agents advancing beyond early-phase trials. For a systematic overview of the evidence level of each candidate, ranging from clinical trial data to preclinical findings, readers are referred to Table 1, where agents are classified according to translational maturity. Many of the therapeutic agents discussed below act on upstream signaling pathways and consequently affect multiple hallmark features. For example, mTOR inhibitors such as rapamycin not only restore defective autophagy but also inhibit angiogenesis and attenuate inflammatory responses [25, 71, 72]. Thus, rather than targeting a single downstream phenotype, these agents modulate upstream hubs that coordinate diverse pathological processes. In evaluating the therapeutic candidates discussed below, it is important to distinguish between evidence of preclinical efficacy, evidence of safety in human populations, and evidence of actual modification of disease natural history.

**Table 1:** Candidate therapeutic interventions for CCMs, classified by evidence level, clinical-trial status, and availability of reported results

Evidence level	Therapeutic candidate	Hallmarks of CCMs	Targeted molecular pathway	Multi-hallmark effects	Translational maturity	Primary endpoint(s)	Ref
I. Clinical trials (status and reported results specified for each entry; see note)	Propranolol	Angiogenesis	Non-selective β-adrenergic receptor antagonist	Also strengthens endothelial junctions	NCT03589014 (Phase 2, completed, results reported in 2023)	New occurrence of clinical events CCM-related, that is ICH and FND excluding seizures. over 24 months	[32, 192, 227–230]
					NCT03474614 (Phase 2, terminated, no results reported)	Global mRNA and miRNA expression changes in blood and CCM tissue	
					NCT03523650 (Phase 1, status unknown since 2018, no results reported)	Number of symptomatic and silent hemorrhages on MRI over 36 months	
	Rapamycin (efficacy on mouse varies by genetic context)	Defective autophagy	mTOR	Also inhibits angiogenesis and attenuates inflammation	NCT06091332 (Phase 2, recruiting, no results reported)	Safety, assessed by serious adverse events over 24 months	[25, 27, 231]
	REC-994 (Tempol)	Oxidative stress	ROS (scavenger of superoxide)	Also reduces inflammation	NCT05085561 (Phase 2, completed, no results reported)	Safety (incidence and severity of adverse events)	[168]
	Atorvastatin	Vascular leakage	RhoA/ROCK (HMG-CoA reductase)	Also reduces inflammation and attenuates EndMT	NCT02603328 (Phase 1/2, completed, results reported in 2025)	Percentage change in mean lesional iron deposition per year, measured by QSM and averaged over 2 years	[232, 233]
	Daratumumab (anti-CD38)	Inflammation and immune response	B cells	—	NCT07026604 (Early Phase 1, not yet recruiting, no results reported)	Safety and preliminary efficacy	—
	Simvastatin	Vascular leakage	RhoA/ROCK (HMG-CoA reductase)	Also reduces inflammation and attenuates EndMT	NCT01764451 (Early Phase 1, terminated, no results reported)	Change in blood–brain barrier permeability over three months measured by dynamic contrast-enhanced MRI (DCEMRI)	[168, 234–236]
II. Observational human data	Aspirin	Inflammation and immune response	COX	Also reduces vascular leakage and antiplatelet	Observational (human)	—	[237–240]
	Clopidogrel	Others	Antiplatelet	Also reduces inflammation	Observational (human)	—	[240]
	Bevacizumab	Angiogenesis	VEGF	—	Observational (human)	—	[241]
III. Preclinical (in vivo) evidence	SU5416 (Semaxanib)	Angiogenesis	VEGFR2	—	Preclinical (in vivo)	—	[117]
	Metoprolol	Angiogenesis	β1-adrenergic receptor antagonist	Also strengthens endothelial junctions	Preclinical (in vivo)	—	[227]
	TSP1 replacement (Full-length TSP1 or 3TSR)	Angiogenesis	TSP1	Also reduces vascular leakage	Preclinical (in vivo)	—	[64]
	Rebastinib	Angiogenesis	Tie2	Also reduces vascular leakage	Preclinical (in vivo)	—	[95]
	ANGPT2 neutralization antibody	Angiogenesis	ANGPT2	Also reduces vascular leakage	Preclinical (in vivo)	—	[96]
	Fasudil	Vascular leakage	ROCK	Also attenuates EndMT	Preclinical (in vivo)	—	[79, 234, 242]
	H-1152	Vascular leakage	ROCK	Also attenuates EndMT	Preclinical (in vivo)	—	[21, 79]
	BA-1049	Vascular leakage	ROCK2	Also attenuates EndMT	Preclinical (in vivo)	—	[243]
	Fluvastatin	Vascular leakage	RhoA/ROCK (HMG-CoA reductase)	Also reduces inflammation and attenuates EndMT	Preclinical (in vivo)	—	[244]
	Bisphosphonate zoledronic acid monohydrate	Vascular leakage	Protein prenylation inhibitor	Also reduces angiogenesis	Preclinical (in vivo)	—	[244]
	β1 integrin monoclonal antibody	Vascular leakage	β1 integrin	Also reduces angiogenesis	Preclinical (in vivo)	—	[245]
	CD5-2	Vascular leakage	miR-27a/VE-cadherin	Also reduces angiogenesis	Preclinical (in vivo)	—	[246]
	Sulindac sulfide	EndMT	TGFβ/β-catenin	Also reduces inflammation	Preclinical (in vivo)	—	[128]
	Sulindac sulfone	EndMT	TGFβ/β-catenin	Also reduces inflammation	Preclinical (in vivo)	—	[128]
	Ponatinib (AP24534)	EndMT	Multikinase (including MEKK3)	Also inhibits angiogenesis	Preclinical (in vivo)	—	[70]
	Indirubin-3-monoxime	EndMT	ERK5-KLF2	Also inhibits angiogenesis	Preclinical (in vivo)	—	[247, 248]
	Multifunctional platinum@BSA-rapamycin nanocarriers	Defective autophagy	mTOR	Also inhibits angiogenesis and attenuates inflammation	Preclinical (in vivo)	—	[249]
	Celecoxib	Inflammation and immune response	COX-2	Also reduces vascular leakage	Preclinical (in vivo)	—	[94]
	13-cis-Retinoic acid	Inflammation and immune response	Anti-inflammatory and anti-tumorigenic effects	Also inhibits angiogenesis	Preclinical (in vivo)	—	[247]
	DNase I	Inflammation and immune response	NETs	Also reduces vascular leakage	Preclinical (in vivo)	—	[142]
	Anti-BR3 antibody	Inflammation and immune response	B cells	—	Preclinical (in vivo)	—	[149]
	Vitamin D3 (cholecalciferol)	Oxidative stress	Vitamin D supplement	Also restores autophagy and reduces inflammation	Preclinical (in vivo)	—	[168]
	N-acetylcysteine (NAC)	Oxidative stress	ROS	Also reduces inflammation	Preclinical (in vivo)	—	[158]
	Tak242 (Resatorvid)	Influenced gut microbiome	TLR4 inhibitors	Also reduces inflammation	Preclinical (in vivo)	—	[66]
	Hypoacetylated LPS	Influenced gut microbiome	Competitively antagonizes TLR4	Also reduces inflammation	Preclinical (in vivo)	—	[66]
	Broad-spectrum antibiotic	Influenced gut microbiome	Microbiome	—	Preclinical (in vivo)	—	[66]
	Blocking antibodies against TM (aTM)	Others	Anti-TM	Also reduces inflammation	Preclinical (in vivo)	—	[189]
	Blocking antibodies against EPCR (rcr252)	Others	Anti-EPCR	Also reduces inflammation	Preclinical (in vivo)	—	[189]
IV. Preclinical (in vitro) evidence	Sorafenib	Angiogenesis	Multikinase inhibitor	—	Preclinical (in vitro)	—	[250]
	ENMD-2076	Angiogenesis	FLT3	—	Preclinical (in vitro)	—	[247]
	NVP-BHG712	Angiogenesis	EphB4	—	Preclinical (in vitro)	—	[251]
	GAP27	Vascular leakage	Connexin 43 gap junction	—	Preclinical (in vitro)	—	[113]
	SB-431542	EndMT	TGF-β/SMAD	—	Preclinical (in vitro)	—	[126]
	LY-364947	EndMT	TGF-β/SMAD	—	Preclinical (in vitro)	—	[126]
	LY-2109761	EndMT	TGF-β/SMAD	—	Preclinical (in vitro)	—	[126]
	DMH1	EndMT	TGF-β/BMP	—	Preclinical (in vitro)	—	[126]
	Dorsomorphin	EndMT	TGF-β/BMP	—	Preclinical (in vitro)	—	[127]
	BIX02189	EndMT	MEK5	—	Preclinical (in vitro)	—	[61]
	XMD17-109	EndMT	ERK5	—	Preclinical (in vitro)	—	[61]
	XMD8-92	EndMT	ERK5	—	Preclinical (in vitro)	—	[63]
	Torin 1	Defective autophagy	mTOR	Also inhibits angiogenesis and attenuates inflammation	Preclinical (in vitro)	—	[72]
	Ridaforolimus	Defective autophagy	mTOR	Also inhibits angiogenesis and attenuates inflammation	Preclinical (in vitro)	—	[247]
	Avenanthramide (YAv)	Oxidative stress	SOD2	Also reduces inflammation	Preclinical (in vitro)	—	[252]
	Pt NPs	Oxidative stress	ROS	Also reduces inflammation	Preclinical (in vitro)	—	[253]
	DL-Erythro-dihydrosphingosine	Others	PKC/phospholipase A2/D inhibitor	—	Preclinical (in vitro)	—	[247]
	DL-Homatropine hydrobromide	Others	Muscarinic acetylcholine receptor antagonist	—	Preclinical (in vitro)	—	[247]
	Bosutinib	Others	MST3/STK24	—	Preclinical (in vitro)	—	[254]
	Saracatinib	Others	MST3/STK24	—	Preclinical (in vitro)	—	[254]
	Danusertib	Others	MST3/STK24	—	Preclinical (in vitro)	—	[254]
	Sunitinib	Others	MST3/STK24	—	Preclinical (in vitro)	—	[254]
	Desatinib	Others	MST3/STK24	—	Preclinical (in vitro)	—	[254]
V. Symptomatic/approved agents (no established disease-modifying effect)	Levetiracetam	Others	Antiepileptic drugs	—	Clinical (approved for epilepsy)	—	[255, 256]
	Carbamazepine	Others	Antiepileptic drugs	—	Clinical (approved for epilepsy)	—	[257]
	Lamotrigine	Others	Antiepileptic drugs	—	Clinical (approved for epilepsy)	—	[257]
	Valproate	Others	Antiepileptic drugs	—	Clinical (approved for epilepsy)	—	[257]

Table 1 lists candidate interventions evaluated in CCM models or patients and is organized by evidence level. Registered clinical trials are reported with their recruitment status and, where available, reported results; trials that are terminated, of unknown status, or not yet recruiting have no reported results and therefore do not constitute evidence of efficacy. Interventions used for symptomatic management (e.g., antiepileptics) are distinguished from candidates with potential disease-modifying activity

#### Anti-angiogenic agents

Anti-angiogenic agents target the aberrant angiogenesis central to CCM initiation, with VEGF as a key mediator. Bevacizumab (anti-VEGF) has shown anecdotal efficacy in radiation-induced CCMs [241], SU5416 (VEGFR2 inhibitor) reduces lesion burden in CCM1-knockout mice [117], and sorafenib abrogates VEGF-mediated sprouting in vitro [250]. Propranolol, a non-selective β-adrenergic receptor blocker/antagonist, has emerged as a leading repurposed candidate, with preclinical studies demonstrating reduced lesion development in zebrafish and mouse models, potentially by diminishing VEGF expression and strengthening EC interactions [192, 227, 228]. The classic β1 selective antagonist metoprolol also exhibits similar effects [227]. A phase 2 pilot RCT confirmed the safety and tolerability of propranolol in fCCMs [32, 229], additional registered trials have been terminated or have unknown status, and its clinical utility for symptomatic CCMs remains to be definitively established. However, the meta-analysis failed to demonstrate a statistically significant protective effect of beta-blockers in preventing ICH or developing FNDs in subjects with CCMs [230], underscoring the gap between safety signals and demonstrable clinical benefit. Other anti-angiogenic therapeutic targets include TSP1 with full-length TSP1 or 3TSR [64], ANGPT2 with neutralization antibody [96], TIE2 with Rebastinib [95], FLT3 with ENMD-2076 [247], and EphB4 with NVP-BHG712 [251] showing preclinical promise in normalizing microvascular structure, yet these agents lack clinical evaluation to date.

#### Repairing vascular permeability

Elevated vascular permeability, driven by hyperactivated RhoA/ROCK signaling and disrupted endothelial junctions, acts as another critical therapeutic target. Fasudil (a ROCK inhibitor acting downstream of RhoA) and H-1152 (ROCK inhibitor) reduce vascular leak and lesion burden in preclinical CCM models [21, 79, 234, 242]. BA-1049, a selective ROCK2 inhibitor (ROCK2 is the predominant isoform in the CNS), diminishes lesion formation and hemorrhage in the mouse models in a dose-dependent manner [243]. Statins (e.g. simvastatin, atorvastatin, fluvastatin) that can inhibit RhoA by reducing its geranylgeranylation, have also been shown benefits in treating CCMs [232, 234–236, 244]. Nonetheless, statin use is reported to be associated with higher intracerebral hemorrhage risk, and in CCM patients with statins for cardiovascular issues, reduced brain permeability is observed in white matter rather than lesions [237, 258]. However, the phase 1/2 AT CASH EPOC trial later found that atorvastatin (80 mg daily) failed to reduce iron deposition or rebleeding in CCM patients with recent symptomatic hemorrhage compared to placebo, despite being well-tolerated [233], highlighting that safety in human populations does not necessarily translate into modification of disease natural history. Antibody-mediated manipulation of β1 integrin halts the progression of acute mouse CCM lesions, an effect also ascribed to inhibition of ROCK activity [245]. Further, MicroRNA-based agent (e.g., Blockmir CD5-2) [246], gap junction modulator (e.g., GAP27) [113], and protein prenylation inhibitor (e.g., N-bisphosphonate zoledronic acid monohydrate) [244] enhance endothelial junction integrity in preclinical models, but their translational potentials are untested.

#### Targeting EndMT

Targeting EndMT that is a critical driver of CCM initiation and progression has yielded preclinical success by virtue of inhibition of the TGF-β/BMP/SMAD and β-catenin pathways. TGF-β/BMP/SMAD pathway inhibitors including SB-431542, LY-364947, LY-2109761, DMH1 and dorsomorphin prevent EndMT in CCM-null ECs [126, 127], while sulindac sulfide/sulfone (β-catenin inhibitors) reduce vascular malformations in CCM3-knockout mice [128]. The MEKK3-KLF2/4 cascade, a core pathway modulated by the CCM complex, is repressed by the multikinase inhibitor ponatinib (which inhibits MEKK3 among other kinases), the MEK5 inhibitor BIX02189, and the ERK5 inhibitor XMD17-109 and XMD8-92, thereby reversing EndMT in vitro [61, 63, 70]. Indirubin-3-monoxime, a natural product, inhibits ERK5-KLF2 signaling across multiple species [247, 248], yet its clinical applicability is unknown. The repurposing of ponatinib, identified through systematic screening, exemplifies a powerful approach to drug discovery.

#### Autophagy restoration

Defective autophagy, a hallmark of LOF mutations in CCM genes, is targeted through mTOR inhibition. The administration of rapamycin (a classic mTORC1 inhibitor) and multifunctional platinum@BSA-rapamycin nanocarriers restored autophagic flux and ameliorated CCM phenotypes [25, 27, 231, 249], while Torin 1 inhibited mTOR signaling even in CCM1-depleted cells [72]. The critical distinction here is that mTOR-independent pro-autophagic agents (e.g. xestospongin B) fail to correct the defect [259], highlighting the specificity of the pathway. However, the response to mTOR inhibition is model-dependent. In a *Pdcd10(Ccm3)*-deficient model without concurrent *Pik3ca* GOF, rapamycin suppressed the emergence of extremely large outlier lesions, but after exclusion of these outliers, high-dose rapamycin significantly increased mean lesion burden, was associated with greater attrition, and did not improve acute or chronic hemorrhage [260]. Collectively, these findings indicate that the response to rapamycin is molecular-context dependent, and mTOR inhibition should not be portrayed as a uniformly promising strategy across all CCM subtypes.

#### Anti-inflammatory and immunomodulatory approaches

Anti-inflammatory and immunomodulatory agents address the low shear stress and chronic inflammation driving lesion growth and instability in CCMs. Sulindac sulfone/sulfide [128], 13-cis-retinoic acid [247], and COX inhibitors (e.g., aspirin, celecoxib) [94, 238] reduce CCM lesion progression in preclinical models, with observational data suggesting aspirin may be associated with lower hemorrhagic risk in CCM patients [239]. Combined aspirin-statin use has been reported to correlate with reduced disease progression in observational studies, such findings require prospective validation [238]. DNase I treatment degrades NETs and attenuates vascular permeability [142], but neutralizing antibody development limits durability. Depletion of B cells with anti-BR3 antibodies can reduce CCM lesion number and size in preclinical models [149]. A clinical trial evaluating B-cell targeting with daratumumab in fCCM has been registered (NCT07026604, Early Phase 1) but has not yet started recruitment, any clinical benefit therefore remains to be established and immunosuppression risks warrant careful consideration.

#### Redox homeostasis and antioxidant strategies

Oxidative stress contributes to CCM pathogenesis through ROS-mediated endothelial dysfunction. Restoring redox homeostasis via antioxidants (e.g. tempol, NAC, YAv) and platinum nanoparticles is capable of alleviating CCM phenotypes in the preclinical models [158, 168, 252, 253]. Importantly, the effects of ROS scavengers appear to be downstream or independent of mTOR activation [72], suggesting they target a parallel pathogenic pathway. Vitamin D3, with its combined antioxidant, anti-inflammatory, and pro-autophagic properties [168, 261, 262], has shown protective effects in a mouse model and represents a candidate for combination therapy [263]. The concept of targeting “oxy-inflammation” is gaining attention, but the clinical evidence for antioxidant monotherapy in chronic diseases has been disappointing [264], implying that combination strategies may be imperative.

#### Gut microbiome manipulation

Gut microbiome dysregulation has emerged as a novel therapeutic axis, with TLR4 as a critical hub between the gut-brain axis and CCM pathogenesis. Resatorvid (TLR4 antagonist) and hypoacetylated LPS reduce CCM lesion volume by 80% and > 90% in mice respectively, while broad-spectrum antibiotics diminish lesion formation by > 95% [66], supporting gut microbiome-based therapies (e.g., fecal transplantation) as promising strategies. Oral administration of P80 (a dietary emulsifier presented in preserved foods) in mice are inclined to disturb colonic mucus barrier, which implicates that common dietary factors including emulsifiers in the processed foods appear to present relevant impacts on the progression of CCMs via detrimental effects on gut barrier [22], highlighting the potential for lifestyle modifications to complement pharmacotherapy, although direct evidence in humans is still lacking.

#### Other potential drugs

Beyond the pathways above, antiepileptic drugs remain the first-line pharmacologic strategy for CCM-related epilepsy (CRE) [265]. Various antiepileptic drugs (e.g. carbamazepine, lamotrigine, valproate and levetiracetam) have been employed to fight against CRE, and they have been shown to be effective and efficient in curbing the occurrence of epileptic seizures, though approximately 40% of patients become refractory [216, 255–257, 266]. The roles of anti-thrombotic therapies are particularly debated. While some evidence suggests antiplatelet agents like aspirin may be safe and even beneficial [267], the coexistence of pro- and anti-coagulatory phenotypes in CCMs complicates clinical decision-making [240, 267, 268]. In addition, systemic administration of blocking antibodies against thrombomodulin or endothelial protein C receptor (EPCR) in mice can in part rescue the bleeding profile [189]. Novel agents identified via pharmacological screening (e.g. DL-erythro-dihydrosphingosine, DL-homatropine hydrobromide) show preclinical efficacy [247], but their mechanisms of action and clinical potential are poorly characterized. Furthermore, various compounds screened out as small-molecule inhibitors for MST3/STK24 may also serve as potential drugs, because MST3/STK24 can form a complex with CCM3 and regulate downstream signaling [254].

In conclusion, the therapeutic landscape for CCMs is at a pivotal juncture. The molecular pathways are increasingly well-defined, providing a pipeline of potential drug targets. However, a critical appraisal reveals that the vast majority of evidence remains preclinical. Among the few agents that have entered clinical trials, none has yet demonstrated definitive modification of the natural history of CCMs. This gap between preclinical promise and clinical validation underscores the need for continued translational research and rigorous trial design. Recent preclinical studies demonstrate that focused ultrasound with microbubbles can significantly enhance the delivery and penetration of model therapeutics ranging from small molecules to larger biologics into both the core and perilesional space of CCMs, offering a promising platform to improve the therapeutic index of future pharmacological agents [269]. The heterogeneity of the disease, both in terms of genetic drivers and clinical presentation, demands a personalized approach and the development of reliable biomarkers for patient stratification and treatment monitoring. Addressing these challenges will be essential for transforming the trajectory of CCM disease for patients.

## Conclusion and future perspectives

It has been widely held that CCMs, which are found in approximately 0.5% of the population, are regarded as the third most common cerebral vascular malformation [6, 9]. CCMs can occur at any age, with a peak incidence between 40 and 60 years (younger in familial cases), and no obvious sex predilection has been established. CCM lesions can be present in the solitary manner for sCCM (80% of cases) or in the multiple manner for fCCM (20%) [9, 40]. Notably, cutaneous and retinal vascular lesions can be observed in around 9% and 5% of fCCM cases respectively. In contrast to familial cases, sporadic CCM lesions are commonly associated with developmental venous anomalies, suggesting that distinct pathogenic mechanisms may underlie the two disease forms [270, 271]. Although CCMs may not be the predominant cause of severe cerebrovascular disease, research on this condition has yielded valuable molecular and genetic insights into vascular development and disease progression.

In line with the multifactorial nature of CCMs, not all carriers of mutations in the same CCM gene develop lesions, and only part of individuals harboring CCM lesions eventually display clinical symptoms [272–274]. The clinical characteristics of fCCM are highly heterogeneous, and the presence of mutations within the same gene does not consistently determine disease severity [5, 141]. Additionally, both initiation age and clinical symptom severity tend to be highly variable among family members harboring the identical genetic mutations, including a unique founder mutation in the *CCM1* gene (Q455X, rs267607203), also accepted as the *CCM1* common Hispanic mutation (KRIT1-CHMQ455X), which is widely distributed among the Hispanic population in the southwestern part of United States [46, 47, 275]. Since each CCM protein possesses multiple interaction partners, it is not surprising that their loss affects numerous signaling pathways and cellular processes. A critical caveat of human CCM sequencing studies is that genetic analysis is restricted to surgically excised lesions, as asymptomatic lesions are rarely resected. This introduces a substantial bias toward more aggressive lesions that may harbor activating mutations such as *PIK3CA*.

A growing body of evidence has elucidated that mutations in the three CCM genes may not be sufficient to give rise to the progression of CCMs, but it requires additional events, including elevated oxidative stress and inflammation that have impacts on EC homeostasis [56]. Consistent with this view, approximately 50%−70% of mutation carriers remain asymptomatic or have only minimal symptoms throughout life. Moreover, disease severity varies even among family members of similar age carrying the same genetic defect, with substantial inter-individual differences in lesion number, size, and susceptibility to intracranial hemorrhage [170]. CCM3-induced familial disease tends to present earlier and with more severe manifestations and greater multisystemic involvement compared to CCM1- or CCM2-associated disease, a feature partly attributed to its association with meningioma [17]. CCM3 is fundamental for the formation of the STRIPAK complex, where it bridges the link between GCKIII kinases and other scaffolding proteins [276, 277]. Additionally, CCM3 participates in the CCM Signaling Complex (CSC), which is assembled around CCM2 as the main bridge that allows the interaction between CCM1 and CCM3 [278, 279]. CCM3 also acts as a crucial component of gut barrier. Collectively, these distinct roles potentially explain the profoundly severe phenotypes observed in patients with LOF mutations in the *CCM3* gene [5, 17]. A deeper understanding of the pathogenic mechanisms and risk factors that influence the onset, progression, and severity of CCMs remains a major academic and clinical challenge, and is essential for identifying novel prognostic markers and pharmacological therapies.

Notably, CCM patients tend to possess a rather unpredictable clinical manifestations, a large number of CCM patients remain asymptomatic whereas some CCM patients exhibit symptoms of seizures or focal hemorrhage with varying degree of aggressiveness [4]. Given the fact that surgery is frequently not applicable, effective and efficient pharmacological therapies are urgently required. Apart from antiepileptic drugs, to which approximately 40% of patients are refractory, no pharmacological strategy has been validated to be clinically effective [280]. Reliable therapies that limit lesion number and growth and reduce hemorrhagic potential are critically needed to change the disease trajectory for both asymptomatic and symptomatic patients. Modulating the disease course could substantially reduce the need for high-risk surgeries and offer a therapeutic option for patients with inoperable lesions. Therefore, an enhanced understanding of the molecular basis of CCM progression is essential for developing patient-tailored therapeutic strategies.

Intriguingly, the multistep pathogenesis of CCM disease involves the progressive acquisition of multiple hallmark biological properties, including excessive angiogenesis, vascular leakage, EndMT, defective autophagy, inflammation and immune dysregulation, oxidative stress, gut microbiome alterations, and disruption of the brain vascular microenvironment, which collectively drive lesion formation and progression. Multiple animal models have been utilized for studying CCMs, among which CCM mouse model is the predominant model for investigating the underlying mechanisms and therapeutic strategies for CCM disease. Current models, including endothelial-specific inducible knockouts of *Ccm1*, *Ccm2*, or *Ccm3*, as well as models incorporating *Map3k3* and *Pik3ca* mutations have provided important tools for studying lesion formation and drug screening [25–27]. Of note, current CCM mouse models inducing EC specific knockout in either one of the three CCM genes contribute to the occurrence of CCM lesions in mouse brains and retinas that resemble human lesions. Although CCM gene heterozygous deletion mice display little or no capability on developing CCM lesions in brains when they stay at a genetically unstable genetic background (*Msh2*^ −/− ^or *Trp53*^ −/− ^), these CCM mice do exhibit more severe lesion burden [21, 23, 281]. Notably, prenatal EC-specific CCM gene knockout results in embryonic lethality at approximately E9.5 [12, 93, 282]. However, peri- or postnatal induction of EC-specific deletion enables the generation of mouse models that survive to adulthood and develop chronic CCM lesions [12, 95]. Nevertheless, more refined models are needed to fully recapitulate the chronic adult-onset course, multi-hit genetic architecture, lesion heterogeneity, and microenvironmental influences of human disease. Importantly, the dysregulated signaling pathways in CCMs provide promising therapeutic targets for developing mechanism-based, patient-tailored therapies.

In summary, we have synthesized current knowledge on the molecular and genetic mechanisms that drive CCM progression and have outlined therapeutic strategies based on distinct pathological hallmarks. Several critical questions remain to be addressed. Why do CCMs preferentially develop in the central nervous system? Does the brain-specific microenvironment confer unique susceptibility, or are there additional tissue-specific modifiers yet to be identified? Are there still undefined molecular signaling pathways involved in CCM pathogenesis? The rapid advancement in the CCM field has brought this disease proximate to translational breakthroughs as well as provided a robust template for effective and efficient strategies to unravel the pathogenesis of CCM disease. In particular, novel pharmacological approaches are urgently required to treat patients with severe symptoms from inoperable or multiple lesions, and to prevent de novo lesion formation in susceptible individuals. Furthermore, identifying unique risk and susceptibility factors that predispose to severe disease, along with reliable biomarkers for disease progression and severity, will enable accurate risk evaluation, early therapeutic intervention, and effective monitoring of treatment outcomes.

## Data Availability

Not applicable.
